# Effects of Drying Methods, Temperature, and Initial Moisture Content on Drying Characteristics, Nutritional Quality, Texture, and Oxidative Stability of Peanuts  

**DOI:** 10.3390/foods15071248

**Published:** 2026-04-06

**Authors:** Lixuan Wei, Ping Li, Yanhong Liu, Yongkang Xie

**Affiliations:** 1 College of Engineering, China Agricultural University, 17 Qinghua Donglu, P.O. Box 194, Beijing 100083, China; wlxpem@163.com; 2 Agricultural Products Processing Research Center, Henan Academy of Agricultural Sciences, Zhengzhou 450002, China; lpself@126.com

**Keywords:** peanut, radio frequency-hot air combined drying, nutritional quality, texture, oxidative stability

## Abstract

This study systematically investigated the combined effects of drying method (mid- and short-wave infrared drying, MSID; hot air drying, HAD; radio frequency-hot air combined drying, RF-HAD), drying temperature (35, 45, 55, 60 °C), and initial wet-basis moisture content (20%, 25%, 30%) on drying characteristics, nutritional quality, texture, and oxidative stability of peanuts. RF-HAD achieved the shortest drying time, followed by MSID and HAD. Protein content remained stable across all treatments. Fat, oleic acid, and total amino acids were significantly affected by all three factors with significant two-way interactions; linoleic acid exhibited significant method × moisture and three-way interactions. Hardness, adhesiveness, springiness, gumminess, and chewiness showed significant three-way interactions, indicating interdependent effects. All samples met national standards for acid value and peroxide value. MSID yielded the lowest acid value and peroxide value immediately after drying, suggesting better initial oxidative quality. Acid value was primarily influenced by method and temperature, with significant two-way interactions, whereas peroxide value showed significant main effects and a highly significant three-way interaction. No single drying condition optimized all quality attributes. RF-HAD excels in drying efficiency and texture enhancement but requires temperature control to limit oxidation; MSID offers superior initial oxidative stability and amino acid retention. Initial moisture content acts as an active variable that modulates the effects of drying method and temperature.

## 1. Introduction

Peanut is one of the world’s most important oilseed and economic crops, valued not only for its high oil content but also for its nutritional richness [1,2]. Peanuts are an excellent source of monounsaturated fatty acids (primarily oleic acid), plant-based protein (containing essential amino acids), dietary fiber, and bioactive micronutrients such as vitamin E, folate, and phytosterols [3,4]. The high oleic acid content contributes to cardiovascular health benefits and improved oxidative stability, while the protein profile supports plant-based dietary patterns [5,6]. These nutritional attributes position peanuts as a key ingredient in functional food development and health-conscious consumption. However, fresh peanuts with high moisture content are highly susceptible to mold, quality deterioration, and oil rancidity after harvest [3]. Therefore, efficient and high-quality drying treatment is an essential step to extend the shelf life of peanuts and maintain their nutritional and commercial value. Traditional hot-air-drying technology is widely used, but it has issues such as long drying time, high energy consumption, and potential nutrient loss and texture deterioration due to prolonged thermal exposure [4]. In recent years, novel drying technologies represented by mid- and short-wave infrared drying and radio frequency drying have garnered significant attention in the agricultural product processing field due to their advantages of high heat transfer efficiency and uniform heating [5,6].

Among the various drying technologies available for agricultural products, hot air drying (HAD) remains the most widely used due to its simplicity and low capital cost. However, HAD relies on convective heat transfer from the surface inward, which often results in a “cold center” phenomenon, where the interior heats slowly, leading to prolonged drying times and potential quality degradation [4]. Mid- and short-wave infrared drying (MSID) operates on the principle of radiative heat transfer. Infrared radiation penetrates the material surface and is absorbed directly by water molecules, generating heat within the product. This mechanism enables rapid temperature rise and more uniform heating compared to convective methods, and has been shown to better preserve color and bioactive compounds in fruits and vegetables [7,8]. Radio frequency (RF) drying utilizes a high-frequency electromagnetic field (typically 13.56, 27.12, or 40.68 MHz) to generate heat through dielectric heating. Polar molecules, particularly water, oscillate in response to the alternating field, producing frictional heat uniformly throughout the material. This volumetric heating effect addresses the “cold center” problem and offers significant advantages for drying thick or dense materials [9,10]. In this study, we employed radio frequency-hot air combined drying (RF-HAD), which synergizes the volumetric heating of RF with the surface convective heating of hot air to further enhance drying efficiency and uniformity [11].

Drying temperature is a critical parameter that governs both drying kinetics and final product quality. In peanut drying studies, temperatures ranging from 35 °C to 70 °C have been commonly investigated [4,12,13,14,15,16]. Lower temperatures (35–45 °C) are typically employed to preserve heat-sensitive nutrients such as unsaturated fatty acids and vitamins, but at the expense of longer drying times and higher energy consumption. Higher temperatures (55–70 °C) accelerate moisture removal and reduce drying time, but may promote lipid oxidation, protein denaturation, and textural deterioration [17,18]. Some studies have reported that drying temperatures exceeding 60 °C can lead to significant increases in acid value and peroxide value, compromising the oxidative stability of peanut oil [19,20]. In this study, we selected 35, 45, 55, and 60 °C to represent a gradient from low- to high-temperature drying, enabling systematic evaluation of the trade-offs between drying efficiency and quality retention.

Over the past decade, several studies have investigated the effects of drying methods and conditions on peanut quality. Qu et al. [19] examined the influence of hot air-drying temperatures (35–55 °C) on peanut oil quality, reporting that higher temperatures increased acid value and peroxide value while decreasing vitamin E content. Zhang et al. [4] compared HAD, microwave drying, and vacuum drying, finding that the drying method significantly affected fatty acid composition and sensory attributes. Xie et al. [21] evaluated MSID, HAD, and RF-HAD on peanut drying kinetics and germination characteristics, demonstrating that RF-HAD achieved the shortest drying time. More recently, Chen et al. [22] reported that different drying techniques led to distinct moisture migration patterns and affected the nutritional profile of peanut pods. Although previous studies have investigated the effects of individual drying methods or temperatures on peanut quality, most have focused on a single quality dimension (e.g., drying kinetics or fat stability) and have rarely examined the combined and interactive effects of drying method, drying temperature, and initial wet basis moisture content on the comprehensive quality profile of high-fat peanuts, including drying characteristics, nutritional components, texture, and oxidative stability in an integrated manner. Moreover, initial moisture content has often been treated as a fixed precondition rather than an active process variable. How it interacts with different drying technologies and temperatures to collectively determine the final product quality remains a critical but underexplored issue. Additionally, the initial moisture content corresponds to different stages of the drying process. By studying different initial moisture content levels, it is possible to analyze whether different drying methods and drying temperatures offer advantages during specific drying stages, thereby providing a theoretical basis for stage drying research on peanuts.

To address these gaps, this study systematically evaluates the combined effects of three key factors—drying method (MSID, HAD, RF-HAD), drying temperature (35, 45, 55, 60 °C), and initial wet basis moisture content (20%, 25%, 30%)—on the drying characteristics, nutritional quality, texture, and oxidative stability of peanuts. The findings are intended to provide a comprehensive theoretical basis for optimizing peanut drying processes and to guide the selection of appropriate drying strategies based on specific quality requirements.

## 2. Materials and Methods

### 2.1. Materials and Reagents

Experimental material: Fresh in-shell peanuts (Xiao Baisha variety) were purchased from a single local market in Zhengzhou, China, in October 2025. All peanuts used in this study were from the same batch to ensure material consistency. After removing foreign matter and immature pods, peanuts with uniform size and without visible damage or physiological disorders were selected. The initial wet basis moisture content of fresh peanuts was determined immediately after cleaning using the oven drying method (105 °C, 24 h) and was found to be 37.2% ± 2.3%. Peanuts were washed with clean water to remove surface dirt, and excess surface water was removed by blotting with paper towels. All samples were stored in sealed polyethylene bags at 4 °C prior to moisture adjustment, and all adjustments and drying experiments were completed within 2 weeks.

Main reagents: Petroleum ether, diethyl ether, isopropanol, phenolphthalein, potassium hydroxide, sodium thiosulfate, etc., from Sinopharm Chemical Reagent Co., Ltd. Shanghai, China, all of analytical grade.

### 2.2. Instruments and Equipment

**Figure 1:**
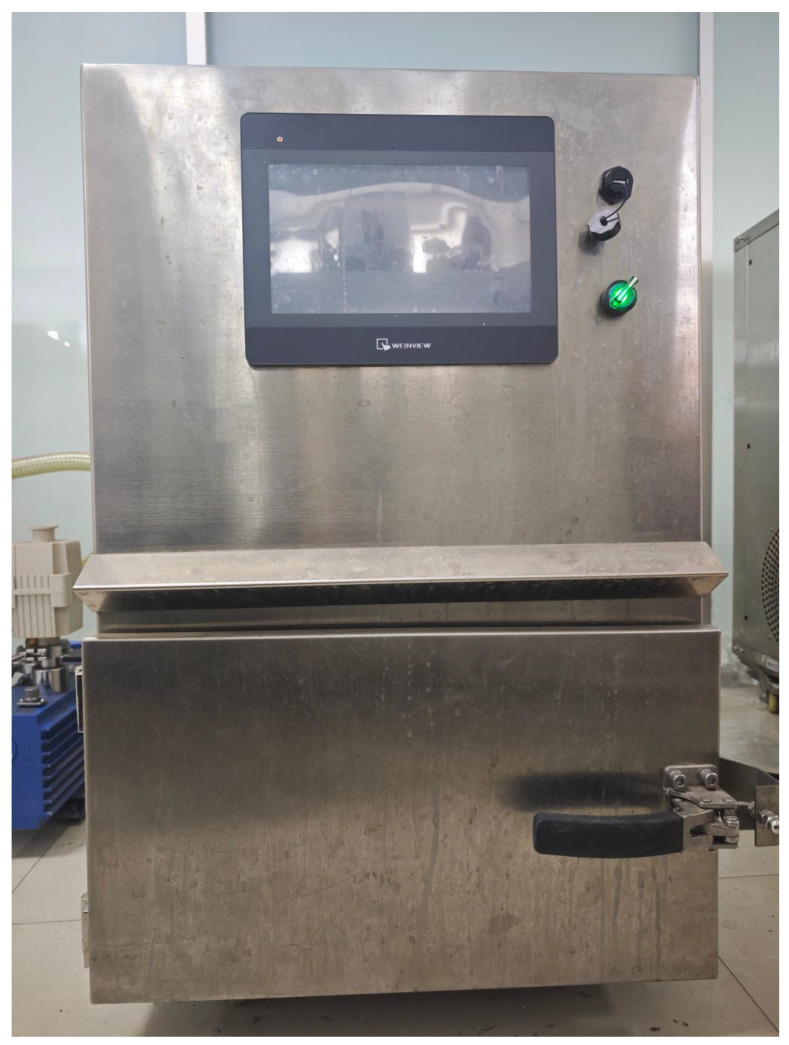
The mid- and short-wave infrared dryer.

The 2100620 mid- and short-wave infrared dryer is shown in Figure 1, manufactured by Suzhou Gaopeng Automation Equipment Co., Ltd, Suzhou, China, with a power of 1920 W and a radiation distance of 10 cm.

**Figure 2:**
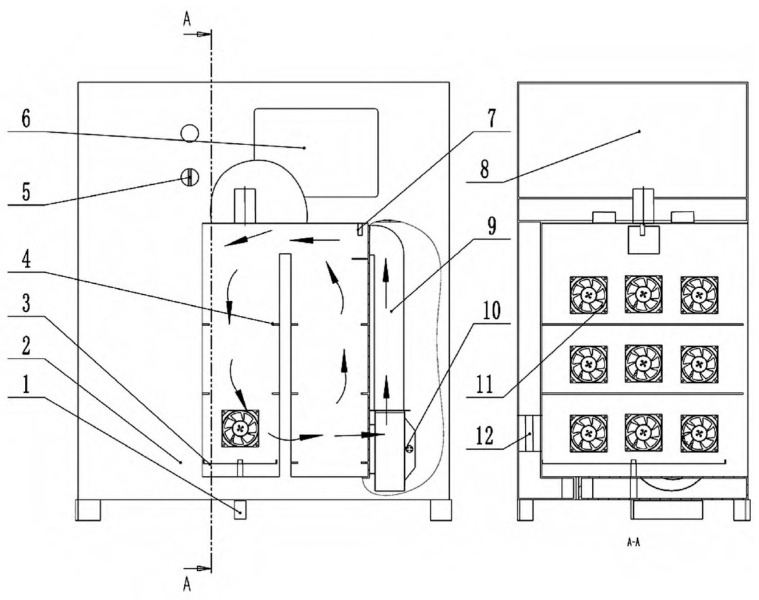
Structure of a hot air dryer. 1. Weighing sensor; 2. Dryer housing; 3. Weighing tray support; 4. Fixed tray support; 5. Switch knob; 6. Touch screen; 7. Temperature and humidity sensor; 8. Distribution cabinet; 9. Heating channel; 10. Centrifugal fan; 11. Spoiler fan; 12. Humidity exhaust fan. Arrow and A‑A indicate the cross‑sectional view.

The structure of the hot air dryer based on temperature and humidity control is shown in Figure 2. Before the operation, the operating temperature and relative humidity are set via the touch screen. Once the drying program is initiated, cold air is heated by the electric heating tubes in the heating channel and blown into the drying chamber by a centrifugal fan. A temperature and humidity sensor (SHT35, Sensirion, Switzerland, measurement error of ±1.5%) is placed inside the drying chamber. When the actual relative humidity exceeds the set value, the exhaust fan is opened; when it falls below the set value, the exhaust fan is closed. The humid and hot air is then disturbed by a disturbance fan inside the drying chamber, circulating between the drying chamber and the heating channel. The internal temperature of the material is measured using an insertion-type temperature sensor (BCL3016P, Bufan Electronics Co., Ltd., Dongguan, measurement error of ±0.3 °C), and the material mass is measured using a load cell (SP4MC3MR, Hottinger Baldwin Measurements, Germany, measurement error of ±0.16%).

**Figure 3:**
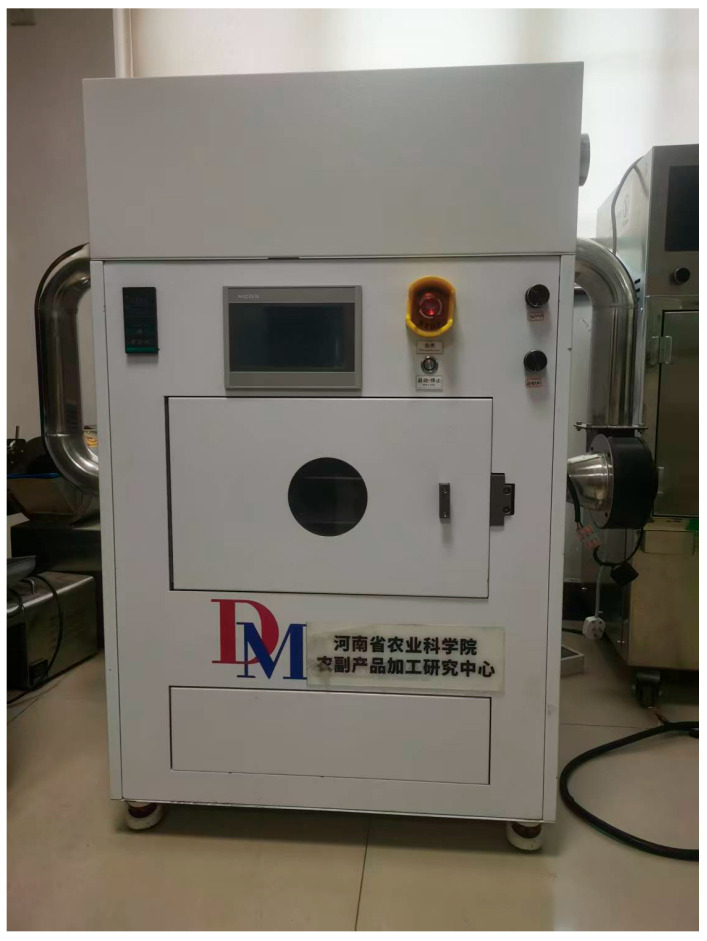
The radio frequency-combined hot air dryer. *The characters ‘河南省农业科学院农副产品加工研究中心’ in the figure refer to the ‘Research Center of Agricultural Products Processing, Henan Academy of Agricultural Sciences’, which is the affiliation of the corresponding author (Yongkang Xie).*

A 6 kW, 27.12 MHz pilot-scale free-running oscillator RF system (SO6B, Strayfield International Limited, Wokingham, UK) with a 6 kW auxiliary hot air system was used for radio frequency combined hot air to dry peanuts. The radio frequency combined hot air dryer is shown in Figure 3. During the drying process, the electrode plate spacing was set to 100 mm.

The drying oven (DHG–9037A) was purchased from Jinghong Experimental Equipment Co., Ltd. (Shanghai, China). The contents of protein, fat, and oleic/linoleic acid were measured using the near-infrared NOVA automatic grain analyzer (DA7250, At–line NIR analysis system, Perten, Denmark). The analytical balance is accurate to 0.001 g (ME430, Mettler–Toledo International Inc., Switzerland) [6].

### 2.3. Experimental Design

The experiment adopted a multi-factor experimental design, primarily selecting three factors: drying method, drying temperature, and initial wet basis moisture content.

Initial wet basis moisture content settings: The initial moisture content of peanuts was adjusted from the fresh value (34.2% ± 0.5% wet basis) to three target levels: 20%, 25%, and 30% (wet basis). Moisture adjustment was performed by spreading peanuts in a single layer in a temperature- and humidity-controlled room (25 °C ± 1 °C). The moisture content was monitored every 2–4 h using a rapid moisture analyzer, which was calibrated against the oven drying method (105 °C, 24 h). When the target moisture content was reached, peanuts were immediately transferred to sealed bags and equilibrated at 4 °C for 24 h to ensure uniform moisture distribution throughout the kernels. This adjustment procedure was applied uniformly to all samples across drying methods and temperature settings.

Drying methods: Three methods were set: mid- and short-wave infrared drying (MSID), hot air drying (HAD), and radio frequency-hot air combined drying (RF-HAD).

Drying temperature: For each drying method, four temperature levels were set: 35, 45, 55, and 60 °C.

Specific experimental combinations totaled 36 groups (3 drying methods × 3 moisture contents × 4 temperatures), with each experiment repeated three times. The specific experimental design is shown in Table 1.

Drying endpoint: All drying experiments were terminated when the wet basis moisture content of peanut kernels reached 10% ± 0.5%. Moisture content during drying was monitored by periodically removing samples, weighing, and verifying the final endpoint using the oven drying method. The consistent endpoint ensures that comparisons of drying time, drying rate, and product quality across different drying methods and conditions are based on the same final moisture target.

### 2.4. Experimental Methods

#### 2.4.1. Drying Characteristic Indicators

All drying experiments were conducted in batch mode. For each experimental condition (drying method × temperature × initial moisture content), approximately 500 g of peanuts were loaded into the drying chamber. During drying, samples were removed from the drying chamber at regular time intervals (every 10–30 min, with shorter intervals for faster drying conditions such as RF-HAD at high temperatures). At each sampling point, approximately 50 g of peanuts were taken from a fixed position within the drying chamber for moisture content determination. Sampling continued until the drying endpoint (wet basis moisture content of 10% ± 0.5%) was reached. Each experimental condition was performed with three independent replicates, and the full sampling procedure was repeated for each replicate. The moisture content of samples at each time point was determined using a rapid moisture analyzer. This instrument was calibrated against the oven drying method (105 °C, 24 h) prior to the experiments to ensure measurement accuracy.

Dry basis moisture content was calculated according to formula (1) [9]:

(1) $$M_{t}=\frac{W_{t}-G}{G}$$

where *W_t_* is the total mass at any drying time, g; *G* is the dry matter mass, g; *M_t_* is the dry basis moisture content, g/g.

Drying rate was calculated according to formula (2) [10]:

(2) $$DR=\frac{M_{t_{1}}-M_{t_{2}}}{t_{1}-t_{2}}$$

where *t*_1_ and *t*_2_ are drying times, h; *M_t_*_1_ and *M_t_*_2_ are the dry basis moisture contents at times t_1_ and t_2_, respectively, g/g; DR is the drying rate between *t*_1_ and *t*_2_, g/(g·h).

Moisture ratio was calculated according to formula (3) [11]:

(3) $$MR=\frac{M_{t}}{M_{0}}$$

where *M*_0_ is the initial dry basis moisture content, g/g; *M_t_* is the dry basis moisture content at time t, g/g.

#### 2.4.2. Nutritional Quality Determination

The dried peanuts were shelled, and peanut kernels of uniform size and without damage were selected. The nutritional quality of the peanut kernels, namely the content of protein, fat, oleic acid, linoleic acid, and amino acids, was determined using an automatic grain analyzer with a peanut quality database (DA7250, At–line NIR analysis system, Perten, Denmark). Operational parameters were as follows: sample weight: 50 ± 1 g; measurement form: non-contact rotary scanning; wavelength range: 950–1650 nm; spectral bandwidth: 7 nm; and spectral resolution: 0.1–10 nm adjustable [6].

#### 2.4.3. Texture Determination

Texture analysis was performed according to the method described by Lu et al. [14] with modifications to ensure standardization and reproducibility. For each treatment group, 15 peanut kernels were randomly selected. After manual shelling, the two cotyledons of each kernel were separated, and any visibly damaged or irregular cotyledons were discarded. From the central region of each cotyledon, a 6 mm × 6 mm × 6 mm cubic piece was cut using a double-blade cutter, with the cutting direction perpendicular to the longitudinal axis of the cotyledon to minimize structural anisotropy. This yielded 30 individual pieces per treatment group (15 kernels × 2 cotyledons). TPA was conducted using the TMS–Pro food texture analyzer equipped with a 75 mm diameter cylindrical probe. The test parameters were set as follows: trigger force 5 N, compression distance 20 mm, pre-test speed 2.0 mm/s, test speed 1.0 mm/s, post-test speed 1.0 mm/s, and compression degree 40% of the original sample height. The two-cycle compression mode was applied to obtain textural parameters. From the force–time curve, the following parameters were calculated using the instrument’s built-in software: hardness (N), adhesiveness (N·s), cohesiveness, springiness, gumminess (N), and chewiness (N·mm). Each of the 30 individual pieces per treatment was measured once, and the reported values represent the mean ± standard deviation (SD) of these 30 replicates.

#### 2.4.4. Acid Value and Peroxide Value Determination

Acid value was determined according to the Chinese national standard [12].

Peroxide value was determined according to the Chinese national standard [13].

### 2.5. Data Analysis

Experimental data were organized using Excel 2021, and graphs were plotted using Origin 2022 software. Three-way ANOVA, one-way ANOVA and Duncan’s multiple range tests were performed using SPSS 21.0, with the significance level set at 0.05. Each group had 3 parallel experiments, and results are expressed as “mean ± standard deviation”.

## 3. Results

### 3.1. Effects of Different Drying Conditions on Peanut Drying Characteristics

#### 3.1.1. Effects of Different Drying Conditions on Peanut Moisture Ratio

**Figure 4:**
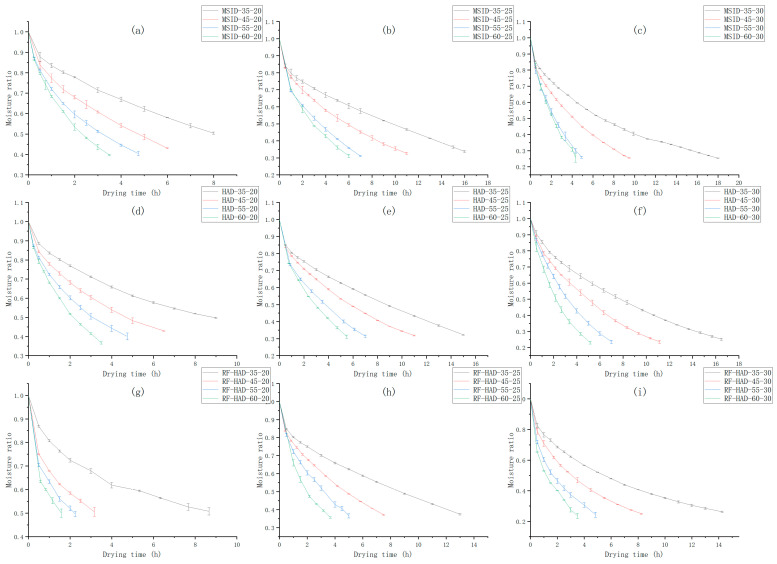
The moisture ratio curves of peanuts under different drying conditions. (**a**) MSID-20%; (**b**) MSID-25%; (**c**) MSID-30%; (**d**) HAD-20%; (**e**) HAD-25%; (**f**) HAD-30%; (**g**) RF-HAD-20%; (**h**) RF-HAD-25%; (**i**) RF-HAD-30%.

As shown in Figure 4, all drying processes exhibited typical falling rate periods, with no obvious constant rate period observed [14]. The drying time of each group is shown in Table 2. Drying temperature had a significant effect on the peanut moisture ratio. The slope of the peanut moisture ratio curve increased with rising drying temperature, and the rate of moisture ratio decrease also accelerated with increasing temperature. As drying time increased, the slope of the moisture ratio curve tended to stabilize [15,16]. When the drying temperature reached 45 °C, the drying time of peanuts significantly shortened [17], indicating that above 45 °C, the kinetic energy and activity of water molecules in peanuts significantly increased, thereby accelerating the moisture migration speed. For all initial wet basis moisture contents, the RF-HAD group at 45 °C showed a significant reduction in drying time compared to 35 °C, averaging a 50.71% reduction, significantly higher than the MSID group (34.49%) and the HAD group (25.16%) [18,19,20]. This suggests that greater involvement of radio frequency energy can significantly accelerate peanut moisture removal.

**Table 2:** Actual drying time (h) to reach the final moisture content (10% wet basis) under different drying conditions.

Temperature/°C	35	45	55	60
Moisture Content/%				
MSID				
20	8.00 ± 0.16	6.00 ± 0.14	4.75 ± 0.11	3.50 ± 0.13
25	16.00 ± 0.00	11.00 ± 0.13	7.00 ± 0.12	6.00 ± 0.12
30	18.00 ± 0.22	9.50 ± 0.11	4.92 ± 0.15	4.33 ± 0.09
HAD				
20	9.00 ± 0.10	6.50 ± 0.12	4.75 ± 0.09	3.50 ± 0.13
25	15.00 ± 0.24	11.00 ± 0.23	7.00 ± 0.11	5.50 ± 0.06
30	16.50 ± 0.30	11.17 ± 0.23	7.00 ± 0.12	5.17 ± 0.08
RF-HAD				
20	8.67 ± 0.13	3.17 ± 0.06	2.25 ± 0.10	1.58 ± 0.05
25	13.00 ± 0.33	7.50 ± 0.20	5.00 ± 0.09	3.67 ± 0.11
30	14.25 ± 0.26	8.25 ± 0.21	4.83 ± 0.16	3.50 ± 0.08

Note: Values represent mean ± SD of three replicates.

Initial wet-basis moisture content also significantly affected the peanut moisture ratio. As the initial moisture content increased, the drying time of peanuts significantly increased, and the trend of change in the moisture ratio curve slope became more gradual.

The drying method also significantly influenced the peanut moisture ratio. Under the same initial moisture content and drying temperature conditions, the drying time of the RF-HAD group was significantly shorter than that of the MSID and HAD groups, especially under conditions of low initial moisture content and high drying temperature. Similar phenomena have also been observed in studies on the drying of other materials [21,22]. For instance, at an initial moisture content of 20% and drying temperature of 60 °C, the drying time of the RF-HAD group was shortened by 54.72% compared to the other two groups. Therefore, the moisture ratio decrease in the RF-HAD group was the fastest among the three drying methods, indicating a more efficient drying process under the same temperature and initial moisture conditions. This is attributed to the volumetric heating effect of radio frequency energy combined with convective heat transfer from hot air, which enhances internal moisture migration and accelerates overall drying. However, direct evidence regarding moisture gradients and crack formation requires further investigation through moisture distribution mapping and microstructural analysis, which will be the focus of future studies.

#### 3.1.2. Effects of Different Drying Conditions on Peanut Drying Rate

**Figure 5:**
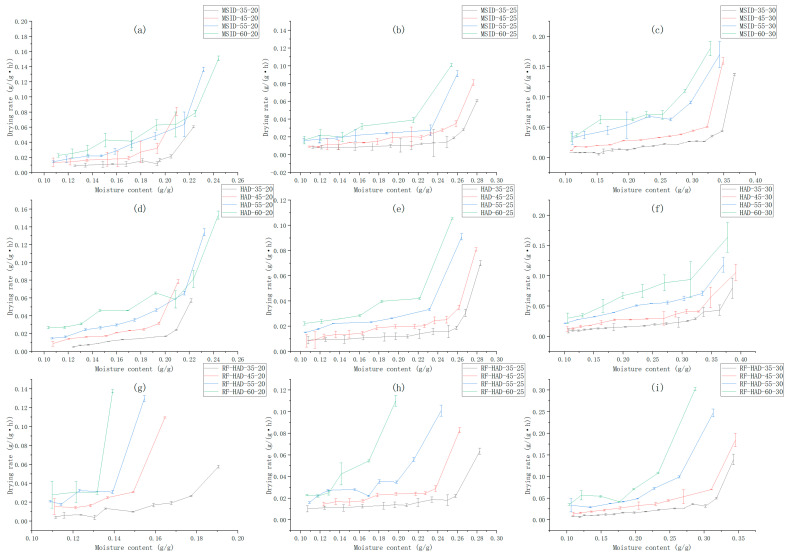
The drying rate curves of peanuts under different drying conditions. The moisture ratio curves of peanuts under different drying conditions. (**a**) MSID-20%; (**b**) MSID-25%; (**c**) MSID-30%; (**d**) HAD-20%; (**e**) HAD-25%; (**f**) HAD-30%; (**g**) RF-HAD-20%; (**h**) RF-HAD-25%; (**i**) RF-HAD-30%.

As shown in Figure 5, the average drying rate of peanuts increased with rising drying temperature. The drying rate accelerated significantly as the temperature increased. In the 55–60 °C range, the increase in drying rate was particularly pronounced, but the magnitude of increase varied with drying method. In the early drying stage, the drying rate decreased significantly, and the influence of drying temperature was more prominent during this phase. The peanut temperature gradually increased, and external moisture was rapidly removed. At this time, the peanut moisture content was relatively high, and moisture loss was relatively fast. Simultaneously, increased drying temperature also accelerated the rate of moisture loss, and internal moisture gradually migrated outward. As drying progressed, the peanut moisture content decreased, becoming mostly bound water, and the rate of internal moisture migration decreased, leading to a slowdown in the drying rate [18,19,20]. Similar phenomena have also been observed in studies on other oil crop seeds [23,24].

Initial wet-basis moisture content significantly affected the drying rate of peanuts. Higher initial moisture content resulted in a faster drying rate in the early stage, but the rate difference diminished in the later stage (low moisture content phase). Higher initial moisture content meant greater kinetic energy and activity of water molecules in the peanuts, leading to relatively faster moisture loss in the early drying stage.

The drying method also significantly affected the drying rate of peanuts. The RF-HAD group exhibited the highest drying rate under all temperature and moisture content conditions, especially at a drying temperature of 60 °C and an initial wet basis moisture content of 30%, where its drying rate was significantly faster than that of the MSID and HAD groups. This is attributed to the synergistic effect of the internal volumetric heating from radio frequency energy and the convective heating from hot air, accelerating internal moisture migration.

### 3.2. Effects of Different Drying Conditions on Peanut Nutritional Quality

The drying process may lead to the loss or alteration of heat-sensitive nutrients. This study primarily focused on protein, fat, fatty acid composition, and amino acid content.

#### 3.2.1. Fat and Fatty Acid Composition

As shown in Table 3, drying temperature was the primary factor affecting fat content and fatty acid composition. Overall, as the drying temperature increased from 35 °C to 60 °C, the measured crude fat content of peanuts showed an upward trend. This apparent increase should be interpreted with caution, as it may reflect a combination of factors rather than a true net increase in fat. First, the substantial loss of moisture at high temperatures leads to concentration of dry matter, resulting in a higher relative percentage of fat [25]. Second, high temperatures may inactivate lipase and other hydrolytic enzymes, potentially reducing enzymatic lipid degradation during drying [26]. Third, thermal treatment can disrupt peanut microstructure, which may enhance the extraction efficiency of fat during NIR analysis, leading to higher predicted values [4]. For example, for HAD-group peanuts with 20% initial moisture content, the fat content at 60 °C (50.34%) increased by 8.4% compared to that at 35 °C (46.46%). It is important to note that all fat content data in this study were obtained using near-infrared spectroscopy, and the absence of a gravimetric reference method validation means that the observed differences should be interpreted as trends in predicted values rather than absolute changes in fat content.

In terms of fatty acid composition, oleic acid content increased with rising drying temperature, while linoleic acid content showed a decreasing trend. For instance, for MSID-group peanuts with 20% initial moisture content, the oleic acid content at 55 °C (31.23%) increased by 24.32% compared to that at 35 °C (25.12%), while linoleic acid decreased from 48.11% to 44.92%, a relative reduction of 6.63%. Linoleic acid is a polyunsaturated fatty acid that is more sensitive to heat; therefore, high temperatures may cause partial oxidation or degradation. As a polyunsaturated fatty acid, linoleic acid exhibits higher chemical reactivity than the monounsaturated oleic acid. Moya Moreno et al., using FTIR technology, confirmed that during thermal oxidation, polyunsaturated fatty acids degrade preferentially through a free radical chain reaction, with their hydroperoxides decomposing into secondary products, leading to a decrease in unsaturation. Recent studies have also indicated that under thermal stress, the content of polyunsaturated fatty acids can decrease by 60–75% [27,28]. Furthermore, the accelerated degradation of the natural antioxidant vitamin E in peanuts under high temperatures weakens the oil’s antioxidant defense system, making unsaturated fatty acids such as linoleic acid more susceptible to oxidative attack.

Linoleic acid is a polyunsaturated fatty acid that is more sensitive to heat, and, therefore, high temperatures may cause partial oxidation or degradation. As a polyunsaturated fatty acid, linoleic acid exhibits higher chemical reactivity than the monounsaturated oleic acid. Moya Moreno et al., using FTIR technology, confirmed that during thermal oxidation, polyunsaturated fatty acids degrade preferentially through a free radical chain reaction, with their hydroperoxides decomposing into secondary products, leading to a decrease in unsaturation [28]. Recent studies have also indicated that under thermal stress, the content of polyunsaturated fatty acids can decrease by 60–75% [27,28]. Furthermore, the accelerated degradation of the natural antioxidant vitamin E in peanuts under high temperatures weakens the oil’s antioxidant defense system, making unsaturated fatty acids such as linoleic acid more susceptible to oxidative attack. Consequently, the observed decrease in linoleic acid content and the relative increase in oleic acid percentage with increasing drying temperature are primarily attributable to the preferential thermal degradation of polyunsaturated fatty acids, which are more susceptible to oxidation than monounsaturated fatty acids. This differential stability results in an increased proportion of oleic acid in the remaining fatty acid profile, rather than a direct conversion of linoleic acid to oleic acid [29].

Fat content varied significantly among treatments (Table 3). Three-way ANOVA (Table 4) revealed significant main effects for M (F_2,72_ = 17.397, *p* < 0.001), T (F_3,72_ = 60.920, *p* < 0.001) and MC (F_2,72_ = 14.718, *p* < 0.001). The interaction M × MC was also significant (F_4,72_ = 16.895, *p* < 0.001), whereas M × T, T × MC and the three-way interaction were not. The significant M × MC interaction indicates that the effect of drying method on fat content depends on the initial moisture content. For example, in the MSID group, increasing MC from 20% to 30% raised fat content at 55 °C, while the opposite trend was observed in the HAD group. Overall, the apparent increase in measured fat content with rising temperature (e.g., HAD 20%: from 46.46% at 35 °C to 50.34% at 60 °C) reflects a combination of moisture loss (concentration of dry matter) and possible changes in extraction efficiency, rather than a true net increase in fat.

Three-way ANOVA for oleic acid (Table 4) showed significant main effects for M (F_2,72_ = 15.067, *p* < 0.001), T (F_3,72_ = 61.374, *p* < 0.001) and MC (F_2,72_ = 23.339, *p* < 0.001), and a significant M × MC interaction (F_4,72_ = 3.001, *p* = 0.024). No other interactions were significant. For linoleic acid (Table 4), main effects of M and T were significant (F_2,72_ = 60.860, *p* < 0.001; F_3,72_ = 23.420, *p* < 0.001), while MC was not (*p* = 0.500). The M × MC interaction (F_4,72_ = 22.127, *p* < 0.001) and the three-way interaction (F_12,72_ = 2.976, *p* = 0.002) were significant. These results confirm that the relative increase in oleic acid and decrease in linoleic acid with increasing temperature are primarily due to the preferential thermal degradation of more highly unsaturated linoleic acid, not a direct conversion between the two. The significant interactions involving MC indicate that the initial moisture content modulates these temperature-dependent changes.

#### 3.2.2. Protein and Amino Acids

Different drying methods and conditions had no significant effect (*p* > 0.05) on peanut protein content. Protein content remained stable within the range of 24–26% across all groups. This indicates that the drying conditions employed in this study did not cause severe protein denaturation or loss.

However, the total amino acid content fluctuated under different conditions. A noteworthy observation was that, under the same initial moisture content (especially 20% and 25%), the amino acid content of HAD-treated samples was significantly lower than that of MSID and RF-HAD samples at most temperatures (Table 5). For example, at an initial moisture content of 20% and drying temperature of 45 °C, the amino acid content of the HAD group was 23.06%, while that of the MSID and RF-HAD groups were 25.34% and 25.10%, respectively. This may be related to the longer duration and more sustained thermal action of hot air drying, potentially leading to Maillard reactions or thermal degradation of some amino acids. The prolonged mild heating of HAD creates ideal conditions for the Maillard reaction. The Maillard reaction involves the reaction between reducing sugars and amino acids (particularly the ε-amino group of lysine), and the reaction products render amino acids unavailable. The prolonged residence time of HAD in the intermediate water activity range facilitates the occurrence of this reaction, leading to a decrease in amino acid content [30]. Notably, Xie et al. found that HAD treatment resulted in the highest protein content but lower amino acid content, indicating that protein retention is not equivalent to amino acid retention. The Maillard reaction and the interconversion of amino acids may lead to unchanged total nitrogen content while the content of specific amino acids decreases [6].

Three-way ANOVA (Table 4) revealed that none of the main effects or interactions were statistically significant for protein content (*p* > 0.05 for all sources). The model showed poor fit (R^2^ = 0.269, adjusted R^2^ = −0.086), indicating that the three factors and their interactions explained only a small portion of the variance in protein content. This confirms that protein content in peanuts remained stable across all drying conditions, consistent with the descriptive statistics presented in Table 3.

Total amino acid content was significantly affected by drying method and initial moisture content, with HAD generally showing lower values than MSID and RF-HAD (Table 3). Three-way ANOVA (Table 4) revealed significant main effects for M (F_2,72_ = 25.598, *p* < 0.001), T (F_3,72_ = 3.749, *p* = 0.015) and MC (F_2,72_ = 22.023, *p* < 0.001). All two-way interactions except M × T (which approached significance, *p* = 0.062) were significant, and the three-way interaction was also significant (F_12,72_ = 2.590, *p* = 0.006). The complex interactions indicate that the retention of amino acids during drying is influenced by the interplay of all three factors, with prolonged mild heating in HAD potentially promoting Maillard reactions and thermal degradation.

### 3.3. Effects of Different Drying Conditions on Peanut Textural Properties

Textural properties are key indicators for evaluating the eating quality of peanuts. This study found that drying method, temperature, and initial wet basis moisture content had significant interactive effects (*p* < 0.05) on the hardness, springiness, chewiness, etc., of peanuts.

#### 3.3.1. Influence of Drying Method and Temperature

Overall, RF-HAD at higher temperatures (55 °C and 60 °C) significantly increased the hardness, gumminess, and chewiness of peanuts. For example, at an initial moisture content of 20% and drying temperature of 60 °C, the hardness (108.92), gumminess (13.66), and chewiness (10.04) of RF-HAD group peanuts were significantly higher than those of the MSID and HAD groups (Table 6). This may be because radio frequency energy rapidly and uniformly heats the peanut interior. Combined with hot air, it promotes rapid vaporization and escape of internal moisture, forming a denser and more porous network structure, thereby enhancing hardness and chewiness. The volumetric heating effect of RF-HAD rapidly and uniformly raises the temperature inside the material. The rapid vaporization of moisture generates steam pressure, which “puffs” the tissue structure, forming a porous network. Research by Xie et al. [6] indicated that the microstructure of peanuts treated with RF-HAD exhibited larger and more numerous pores, whereas hot air drying resulted in a dense structure. This porous architecture can withstand greater loads upon force application and undergo more deformation before rupture, thus exhibiting higher hardness and chewiness. Secondly, high temperatures (55–60 °C) induce the thermal denaturation of proteins. Studies by Guo et al. [31] confirmed that RF-HAD treatment increased the β-sheet content in peanut protein by 41.39% while decreasing the α-helix content by 9.32%. The β-sheet structure is more stable and rigid than the α-helix; its increased content signifies enhanced rigidity of the protein network structure, directly contributing to the increase in hardness and chewiness.

In contrast, HAD generally produced peanuts with a relatively softer texture and lower springiness under most conditions. For instance, at an initial moisture content of 20% and drying temperature of 35 °C, the springiness (0.30) of HAD group peanuts was significantly lower than that of MSID (0.39) and RF-HAD (0.42) (Table 6). The textural properties of mid- and short-wave infrared drying (MSID) typically fell between the two but exhibited higher adhesiveness under specific conditions (e.g., low-temperature drying of high-moisture samples)

#### 3.3.2. Influence of Initial Wet Basis Moisture Content

Initial wet basis moisture content significantly influenced the textural properties after drying. Higher initial moisture content (30%) tended to result in higher hardness, springiness, and chewiness after drying under most drying methods. For example, in the MSID group at a drying temperature of 60 °C, the hardness (92.50) and chewiness (6.29) of peanuts with 30% initial moisture content were significantly higher than those of samples with 20% and 25% moisture content (Table 7). Dean et al. [32], using scanning electron microscopy to compare the microstructure of peanuts with high (7%) and low (4%) initial moisture content after heat treatment, revealed an important pattern: high moisture content samples exhibited more severe surface damage due to steam escape, but their internal cellular structure was protected by the presence of water; conversely, low moisture content samples showed less surface damage, but their internal cellular components were more distorted and denser. This implies that low moisture content samples may have undergone ‘internal collapse’, leading to damaged cellular networks and reduced springiness. Simultaneously, high moisture content samples required a longer heating time to reach the drying endpoint; while this increased surface damage, the internal structure remained relatively intact due to the buffering effect of water. Furthermore, research by Zhu et al. [33] observed that the hardness of peanut kernels during drying exhibited a dynamic trend of ‘initial increase, subsequent decrease, then increase again’, indicating that high moisture content samples formed a denser structure through shrinkage during the later stages of drying. These factors collectively contribute to the higher hardness, springiness, and chewiness observed in peanuts with higher initial moisture content after drying.

However, there were exceptions to this trend. In the RF-HAD group at lower drying temperatures (35 °C), peanuts with 30% initial moisture content had the lowest hardness (42.09) (Table 7). This suggests that for RF-HAD, excessively high initial moisture content at low temperatures may not yet form a strong and tough structure through effective energy coupling; the specific mechanism requires further study.

#### 3.3.3. Summary of Factorial Effects for Texture

Three-way ANOVA results for each parameter are summarized in Table 8.

Hardness: All three main effects were highly significant (M: F_2,144_ = 42.058, *p* < 0.001; T: F_3,144_ = 156.042, *p* < 0.001; MC: F_2,144_ = 35.135, *p* < 0.001). All two-way interactions and the three-way interaction were also significant (*p* < 0.001 for all). These results confirm that the effect of drying method on hardness depends simultaneously on temperature and initial moisture content. For instance, RF-HAD at 60 °C produced the hardest peanuts (108.92 N) when the initial MC was 20%, while at 35 °C the same method gave relatively lower hardness (61.18 N). Higher initial MC (30%) generally increased hardness, but the effect was method-dependent.

Cohesiveness: All main effects were significant (M: F_2,144_ = 20.341, *p* < 0.001; T: F_3,144_ = 9.450, *p* < 0.001; MC: F_2,144_ = 19.215, *p* < 0.001). Among interactions, only M × MC was significant (F_4,144_ = 2.731, *p* = 0.031). Thus, while each factor independently affects cohesiveness, the combined effect is largely additive except for the method-moisture interaction.

Adhesiveness: All main effects and all interactions (including three-way) were highly significant (*p* < 0.001 for all). The model exhibited excellent fit (R^2^ = 0.953). The extremely high F-value for M × MC (422.348) indicates that the adhesiveness of peanuts is strongly determined by the combination of drying method and initial moisture content.

Springiness: All main effects and all interactions were significant (M: F_2,144_ = 65.037, *p* < 0.001; T: F_3,144_ = 27.940, *p* < 0.001; MC: F_2,144_ = 4.607, *p* = 0.011; all interactions *p* ≤ 0.001). The significant three-way interaction confirms that the springiness of dried peanuts depends on the complex interplay of all three factors.

Gumminess: Main effects of M, T and MC were significant (*p* < 0.001). The M × T and M × MC interactions were significant (*p* < 0.001), while T × MC was not (*p* = 0.083). The three-way interaction was significant (*p* = 0.004). Thus, the effect of temperature on gumminess does not depend strongly on initial moisture content, but the other interactions are important.

Chewiness: All main effects and all interactions were significant (M: F_2,144_ = 61.246, *p* < 0.001; T: F_3,144_ = 54.935, *p* < 0.001; MC: F_2,144_ = 17.826, *p* < 0.001; all interactions *p* ≤ 0.022). The significant three-way interaction indicates that chewiness is governed by the combined effects of all three factors.

Overall, the texture results demonstrate that hardness, adhesiveness, springiness, gumminess and chewiness are highly sensitive to the interactive effects of drying method, temperature and initial moisture content, while cohesiveness is mainly influenced by main effects.

### 3.4. Effects of Different Drying Conditions on Peanut Oxidative Stability

Acid Value (AV) measures the amount of free fatty acids released by the hydrolysis of fats by enzymes, oxygen, or microorganisms, while Peroxide Value (POV) indicates the degree of oxidative deterioration of fats and oils. Higher POVs indicate increased peroxide formation during drying, suggesting greater fat deterioration. AV and POV are important indicators for measuring oil rancidity and primary oxidation products.

#### 3.4.1. Positive Effects of Temperature and Initial Moisture Content

As shown in Table 9, higher drying temperatures led to higher acid value and peroxide value in peanuts. Regardless of the drying method used, the AV and POV of samples dried at 60 °C were significantly higher than those dried at 35 °C. High temperatures significantly accelerated the processes of oil hydrolysis and oxidation. Research by Qu et al. [25] found that when the hot air-drying temperature exceeded 45 °C, the acid value and peroxide value of peanut oil increased significantly. Studies by Zhang et al. [4] also confirmed that mechanical drying led to an increase in the acid value and peroxide value of peanut oil. The mechanism underlying this temperature effect can be explained at multiple levels. From the perspective of chemical reaction kinetics, elevated temperatures accelerate the free radical chain reaction of lipids, causing the generation rate of hydroperoxides to increase exponentially. Simultaneously, high temperatures lead to the accelerated degradation of the natural antioxidant vitamin E in peanuts. Qu et al. [25] discovered that when the drying temperature exceeded 50 °C, the vitamin E content decreased significantly, weakening the oil’s antioxidant defense system. Furthermore, there is a synergistic effect between moisture and temperature. Guo et al. [31] pointed out that water, as a polar solvent, promotes the diffusion of oxygen into the oil, activates lipoxygenase, and facilitates the oxidation of unsaturated fatty acids, thereby leading to increased peroxide value and acid value. Research by Luo et al. [26] on oil-tea camellia seeds also confirmed that heat treatment at 60 °C significantly reduced lipoxygenase activity, illustrating the dynamic balance between enzymatic and non-enzymatic oxidation under high-temperature conditions.

Simultaneously, higher initial moisture content also tended to lead to higher oxidation indicators. For example, in the RF-HAD group at a drying temperature of 60 °C, the acid value (0.90) of peanuts with 30% initial moisture content was significantly higher than that of samples with 20% (0.58) and 25% (0.78) initial moisture content (Table 10). Research by Chen et al. [34] indicates that all drying methods led to an increase in the acid value of peanuts (*p* < 0.05), confirming that the drying process itself triggers lipid hydrolysis. A higher initial moisture content implies that more water is directly involved in the hydrolysis reaction of triacylglycerols, generating free fatty acids. Simultaneously, water activity plays a regulatory role in enzyme activity. Silva et al. [35] found in their study on soybeans that lipid and protein degradation were faster in kernels with higher moisture content (14%), and that excessive moisture promoted enzyme activity and microbial development, leading to a significant increase in oil acidity. This mechanism is equally applicable during the peanut drying process—samples with high initial moisture content have high water activity in the early drying stage, resulting in stronger lipase activity and a more intense catalytic hydrolysis reaction.

#### 3.4.2. Comparison of Drying Methods

In terms of controlling oxidative stability, MSID performed best overall. Under most comparable conditions (especially at medium and high temperatures), the AV and POV of MSID group samples were lower than or equivalent to those of the HAD and RF-HAD groups. For instance, when processing peanuts with different initial moisture contents at a drying temperature of 55 °C, the acid values of the MSID group were generally lower than those of the HAD and RF-HAD groups (Table 11). Infrared drying can better preserve natural antioxidants. In their study on oil-tea camellia seeds, Luo et al. [26] found that the α-tocopherol and γ-tocopherol contents in seeds dried by infrared-hot air combined drying were significantly higher than those in seeds dried by hot air alone, and they exhibited better oxidative stability. Research on perilla seeds by Sundar et al. [36] also confirmed that appropriate infrared pretreatment conditions resulted in the highest tocopherol content. These endogenous antioxidants can effectively scavenge free radicals and interrupt the lipid oxidation chain reaction, thereby reducing the peroxide value. Secondly, infrared drying is more efficient at inhibiting enzymes related to lipid oxidation. Research by Luo et al. [26] revealed a key mechanism: infrared treatment significantly reduced lipoxygenase and lipase activities, while simultaneously increasing the sulfhydryl group content and decreasing the α-helix content of the enzyme proteins. This indicates that the alteration in the secondary structure of the enzyme proteins is a significant reason for their decreased activity. This structural inactivation of enzymes is more thorough than simple thermal deactivation.

RF-HAD posed the highest oxidative risk when processing high-moisture samples at high temperatures (60 °C), with its acid value reaching 0.90 (Table 11). The review by Lian [37] clearly points out that radio frequency heating has an inherent drawback of edge overheating. Under high moisture content conditions (30%), water, as a strongly polar molecule, moves vigorously, which may further exacerbate the edge overheating effect, forming hotspot areas and accelerating lipid oxidation. Furthermore, the lipidomics study by Peng et al. [38] confirms that during radio frequency treatment, the hydrolysis of fatty acyl chains and their oxidation into secondary oxides constitute the core behavior of lipid transformation. This process is amplified under conditions of high temperature and high moisture content, leading to the generation of a large number of differential lipids, ultimately manifested as a significant increase in acid value and peroxide value.

#### 3.4.3. Summary of Factorial Effects for Oxidative Stability

The measured acid values ranged from 0.23 to 0.90 mg KOH/g (Table 9), all well below the national standard limit (≤2.5 mg KOH/g). Three-way ANOVA (Table 12) revealed significant main effects for M (F_2,36_ = 25.188, *p* < 0.001) and T (F_3,36_ = 134.173, *p* < 0.001), while MC was not significant (F_2,36_ = 1.138, *p* = 0.332). The M × T (F_6,36_ = 2.925, *p* = 0.020) and M × MC (F_4,36_ = 15.950, *p* < 0.001) interactions were significant, whereas T × MC and the three-way interaction were not. The significant M × T interaction indicates that the increase in AV with temperature differs among drying methods; for example, RF-HAD showed a steeper rise at 60 °C than MSID. The M × MC interaction suggests that the effect of initial moisture content on AV depends on the drying method.

Peroxide values ranged from 0.01 to 0.07 g/100 g (Table 9), also far below the standard limit (≤0.25 g/100 g). Three-way ANOVA (Table 12) showed that all three main effects were highly significant (M: F_2,36_ = 69.135, *p* < 0.001; T: F_3,36_ = 223.146, *p* < 0.001; MC: F_2,36_ = 212.817, *p* < 0.001). All two-way interactions and the three-way interaction were also highly significant (*p* < 0.001 for all). These results demonstrate that the formation of primary oxidation products (hydroperoxides) during drying is highly sensitive to the combined effects of drying method, temperature and initial moisture content.

While all dried peanut samples met the national safety requirements, the statistical analysis reveals distinct mechanisms: hydrolytic rancidity (AV) was mainly driven by drying method and temperature, with interactive effects involving initial moisture content; primary oxidation (POV) was influenced by all three factors and their complex interactions. The lower AV and POVs observed for MSID under most conditions suggest that this method better preserves the initial oxidative quality, possibly due to more efficient inactivation of lipolytic enzymes and better retention of natural antioxidants. However, it is important to note that these measurements reflect the initial oxidation state immediately after drying; the long-term oxidative stability during storage cannot be inferred from these data alone. Further storage studies are needed to determine whether the observed differences translate into extended shelf life.

## 4. Conclusions

This study systematically evaluated the combined effects of drying method (MSID, HAD, RF-HAD), temperature (35–60 °C), and initial moisture content (20–30% w.b.) on drying characteristics, nutritional quality, texture, and oxidative stability of peanuts using a three-factor factorial design with three-way ANOVA. RF-HAD achieved the shortest drying time, followed by MSID and HAD. Protein content remained stable across all treatments. Fat, oleic acid, and total amino acids were significantly affected by all three factors with significant two-way interactions; linoleic acid exhibited significant method × moisture and three-way interactions. The relative increase in oleic acid and decrease in linoleic acid with rising temperature reflect preferential degradation of polyunsaturated fatty acids. HAD resulted in the greatest amino acid loss. Hardness, adhesiveness, springiness, gumminess, and chewiness showed significant three-way interactions, indicating interdependent effects. All samples met national standards for acid value (AV) and peroxide value (PV). MSID yielded the lowest AV and PV immediately after drying, suggesting better initial oxidative quality. AV was primarily influenced by method and temperature, with significant method × temperature and method × moisture interactions, whereas PV showed significant main effects and a highly significant three-way interaction. No single drying condition optimized all quality attributes. RF-HAD excels in drying efficiency and texture but requires temperature control to limit oxidation; MSID offers superior initial oxidative stability and amino acid retention. Initial moisture content acts as an active variable that modulates the effects of drying method and temperature.

Future research could further explore the correlation mechanisms between changes in microstructure (e.g., porosity, cell morphology) during drying and macroscopic texture/oxidation rates, and conduct storage tests to verify the shelf life of products from different drying processes.

## Figures and Tables

**Table 1:** Experimental Design and Parameter Settings.

Experimental Number	Drying Method	Drying Temperature (°C)	Moisture Content (%)
1	MSID	35	20
2	MSID	35	25
3	MSID	35	30
4	MSID	45	20
5	MSID	45	25
6	MSID	45	30
7	MSID	55	20
8	MSID	55	25
9	MSID	55	30
10	MSID	60	20
11	MSID	60	25
12	MSID	60	30
13	HAD	35	20
14	HAD	35	25
15	HAD	35	30
16	HAD	45	20
17	HAD	45	25
18	HAD	45	30
19	HAD	55	20
20	HAD	55	25
21	HAD	55	30
22	HAD	60	20
23	HAD	60	25
24	HAD	60	30
25	RF-HAD	35	20
26	RF-HAD	35	25
27	RF-HAD	35	30
28	RF-HAD	45	20
29	RF-HAD	45	25
30	RF-HAD	45	30
31	RF-HAD	55	20
32	RF-HAD	55	25
33	RF-HAD	55	30
34	RF-HAD	60	20
35	RF-HAD	60	25
36	RF-HAD	60	30

**Table 3:** Effect of drying temperature on the nutritional quality of peanuts.

Temperature/°C	Protein/%	Fat/%	Oleic Acid/%	Linoleic Acid/%	Total Amino Acids/%
MSID-20%					
35	25.09 ± 0.76 ^a^	47.41 ± 0.75 ^c^	25.12 ± 0.97 ^b^	48.11 ± 1.48 ^a^	25.58 ± 0.65 ^a^
45	25.59 ± 0.23 ^a^	48.36 ± 0.14 ^bc^	26.30 ± 0.22 ^b^	46.89 ± 0.86 ^a^	25.34 ± 0.22 ^a^
55	25.22 ± 0.19 ^a^	49.26 ± 0.51 ^ab^	31.23 ± 0.82 ^a^	44.92 ± 0.40 ^b^	25.45 ± 0.17 ^a^
60	25.45 ± 0.90 ^a^	49.95 ± 0.56 ^a^	30.11 ± 0.53 ^a^	44.35 ± 0.24 ^b^	25.39 ± 0.76 ^a^
MSID-25%					
35	25.67 ± 0.55 ^a^	45.59 ± 0.47 ^c^	24.95 ± 0.33 ^d^	49.60 ± 0.43 ^a^	25.42 ± 0.99 ^a^
45	25.18 ± 0.99 ^a^	47.23 ± 0.39 ^b^	26.13 ± 0.42 ^c^	48.73 ± 0.43 ^a^	25.20 ± 0.38 ^a^
55	25.18 ± 0.78 ^a^	47.70 ± 1.03 ^ab^	27.53 ± 0.73 ^b^	48.31 ± 0.47 ^ab^	25.60 ± 0.46 ^a^
60	24.98 ± 1.21 ^a^	49.23 ± 0.99 ^a^	28.91 ± 0.62 ^a^	46.62 ± 1.59 ^b^	25.77 ± 1.10 ^a^
MSID-30%					
35	24.53 ± 0.70 ^a^	46.40 ± 0.81 ^b^	24.50 ± 1.03 ^b^	49.39 ± 0.61 ^a^	25.37 ± 0.59 ^b^
45	25.42 ± 0.47 ^a^	47.21 ± 0.29 ^ab^	25.00 ± 1.21 ^b^	50.12 ± 1.19 ^a^	26.15 ± 0.41 ^ab^
55	25.32 ± 0.31 ^a^	48.67 ± 0.65 ^a^	27.89 ± 1.46 ^a^	49.32 ± 1.07 ^a^	26.86 ± 0.22 ^a^
60	24.49 ± 0.86 ^a^	48.69 ± 1.31 ^a^	29.31 ± 1.61 ^a^	48.22 ± 0.39 ^a^	25.31 ± 0.72 ^b^
HAD-20%					
35	25.90 ± 0.24 ^a^	46.46 ± 0.59 ^d^	25.49 ± 0.99 ^c^	47.01 ± 1.47 ^ab^	23.09 ± 0.56 ^a^
45	25.67 ± 0.26 ^a^	48.19 ± 0.28 ^c^	27.48 ± 0.65 ^b^	47.94 ± 0.94 ^a^	23.06 ± 0.89 ^a^
55	25.76 ± 0.46 ^a^	49.45 ± 0.42 ^b^	29.87 ± 0.86 ^a^	46.97 ± 1.33 ^ab^	23.30 ± 0.73 ^a^
60	25.64 ± 0.05 ^a^	50.34 ± 0.20 ^a^	30.98 ± 1.21 ^a^	44.69 ± 0.86 ^b^	23.50 ± 0.50 ^a^
HAD-25%					
35	25.29 ± 0.26 ^a^	47.75 ± 0.63 ^b^	23.68 ± 1.01 ^b^	44.94 ± 0.70 ^a^	25.42 ± 0.22 ^a^
45	24.90 ± 1.01 ^a^	47.99 ± 0.90 ^ab^	24.92 ± 0.19 ^b^	45.24 ± 1.80 ^a^	25.08 ± 0.89 ^a^
55	25.67 ± 0.95 ^a^	48.87 ± 0.64 ^ab^	28.42 ± 2.60 ^a^	44.49 ± 0.60 ^ab^	25.86 ± 0.81 ^a^
60	25.46 ± 0.68 ^a^	49.33 ± 0.50 ^a^	27.84 ± 0.66 ^a^	42.58 ± 0.69 ^b^	25.33 ± 0.14 ^a^
HAD-30%					
35	24.97 ± 0.37 ^a^	47.24 ± 0.64 ^c^	24.72 ± 0.96 ^b^	46.84 ± 0.66 ^a^	25.37 ± 0.29 ^a^
45	24.45 ± 0.85 ^a^	47.98 ± 0.17 ^bc^	24.92 ± 1.22 ^b^	45.94 ± 0.85 ^ab^	24.72 ± 0.75 ^a^
55	24.99 ± 0.16 ^a^	49.03 ± 0.62 ^ab^	28.42 ± 0.68 ^a^	44.38 ± 1.11 ^b^	25.37 ± 0.17 ^a^
60	25.27 ± 0.43 ^a^	50.05 ± 0.77 ^a^	28.95 ± 1.30 ^a^	44.01 ± 1.41 ^b^	24.58 ± 0.41 ^a^
RF-HAD-20%					
35	24.91 ± 1.40 ^a^	46.09 ± 1.09 ^b^	24.67 ± 2.03 ^a^	46.83 ± 1.40 ^a^	25.93 ± 1.17 ^a^
45	25.13 ± 0.19 ^a^	47.18 ± 1.37 ^ab^	25.88 ± 4.28 ^a^	50.29 ± 2.53 ^a^	25.10 ± 0.15 ^ab^
55	25.49 ± 0.68 ^a^	48.98 ± 0.76 ^a^	30.29 ± 3.14 ^a^	48.42 ± 1.73 ^a^	23.66 ± 0.53 ^b^
60	25.48 ± 1.02 ^a^	48.48 ± 1.27 ^ab^	28.50 ± 1.59 ^a^	48.45 ± 0.84 ^a^	25.38 ± 0.86 ^a^
RF-HAD-25%					
35	25.38 ± 0.43 ^a^	46.40 ± 0.11 ^b^	20.40 ± 1.20 ^b^	49.66 ± 0.59 ^a^	25.00 ± 0.39 ^a^
45	25.67 ± 0.65 ^a^	49.20 ± 0.82 ^a^	26.38 ± 0.70 ^a^	47.75 ± 0.64 ^ab^	23.28 ± 0.54 ^b^
55	25.65 ± 0.37 ^a^	49.53 ± 0.57 ^a^	27.13 ± 1.28 ^a^	47.92 ± 1.05 ^ab^	25.20 ± 0.28 ^a^
60	25.79 ± 1.23 ^a^	49.93 ± 1.36 ^a^	28.46 ± 2.07 ^a^	47.29 ± 1.33 ^b^	25.04 ± 1.09 ^a^
RF-HAD-30%					
35	25.23 ± 1.16 ^a^	45.61 ± 0.90 ^a^	21.71 ± 1.99 ^a^	49.55 ± 1.49 ^a^	24.84 ± 0.97 ^b^
45	25.58 ± 0.20 ^a^	45.27 ± 0.89 ^a^	23.65 ± 1.25 ^a^	47.10 ± 0.93 ^b^	24.99 ± 0.18 ^b^
55	25.35 ± 1.19 ^a^	45.87 ± 1.29 ^a^	24.96 ± 2.25 ^a^	46.40 ± 0.90 ^b^	26.81 ± 1.03 ^a^
60	25.45 ± 0.32 ^a^	47.12 ± 0.48 ^a^	24.15 ± 0.97 ^a^	45.53 ± 1.08 ^b^	26.55 ± 0.24 ^a^

Note: The different lowercase letters indicate a statistically significant difference at *p* < 0.05.

**Table 4:** Three-way ANOVA results for the nutritional quality of peanuts.

Source	Type III Sum of Squares	df	Mean Square	F-Value	*p*-Value
Fat content (%)					
Corrected Model	211.420	35	6.041	9.907	0.000
Intercept	248,835.840	1	248,835.840	408,120.268	0.000
Drying method (M)	21.215	2	10.607	17.397	0.000
Drying temperature (T)	111.432	3	37.144	60.920	0.000
Initial moisture content (MC)	17.947	2	8.974	14.718	0.000
M × T	0.899	6	0.150	0.246	0.959
M × MC	41.205	4	10.301	16.895	0.000
T × MC	5.058	6	0.843	1.383	0.233
M × T × MC	13.665	12	1.139	1.868	0.053
Error	43.899	72	0.610		
Total	249,091.159	108			
Corrected Total	255.319	107			
Note: R^2^ = 0.828, adjusted R^2^ = 0.744.					
Oleic acid content (%)					
Corrected Model	688.308	35	19.666	8.734	0.000
Intercept	76,611.849	1	76,611.849	34,022.870	0.000
Drying method (M)	67.853	2	33.926	15.067	0.000
Drying temperature (T)	414.603	3	138.201	61.374	0.000
Initial moisture content (MC)	105.108	2	52.554	23.339	0.000
M × T	15.317	6	2.553	1.134	0.352
M × MC	27.026	4	6.756	3.001	0.024
T × MC	18.908	6	3.151	1.400	0.227
M × T × MC	39.492	12	3.291	1.462	0.159
Error	162.128	72	2.252		
Total	77,462.284	108			
Corrected Total	850.435	107			
Note: R^2^ = 0.809, adjusted R^2^ = 0.717					
Linoleic acid content (%)					
Corrected Model	405.784	35	11.594	9.502	0.000
Intercept	239,368.844	1	239,368.844	196,179.255	0.000
Drying method (M)	148.518	2	74.259	60.860	0.000
Drying temperature (T)	85.728	3	28.576	23.420	0.000
Initial moisture content (MC)	1.710	2	0.855	0.701	0.500
M × T	5.605	6	0.934	0.766	0.599
M × MC	107.995	4	26.999	22.127	0.000
T × MC	12.659	6	2.110	1.729	0.127
M × T × MC	43.569	12	3.631	2.976	0.002
Error	87.851	72	1.220		
Total	239,862.479	108			
Corrected Total	493.635	107			
Note: R^2^ = 0.822, adjusted R^2^ = 0.736					
Protein content (%)					
Corrected Model	13.906	35	0.397	0.758	0.815
Intercept	69,195.516	1	69,195.516	132,035.013	0.000
Drying method (M)	1.136	2	0.568	1.084	0.344
Drying temperature (T)	0.491	3	0.164	0.313	0.816
Initial moisture content (MC)	2.743	2	1.372	2.617	0.080
M × T	2.564	6	0.427	0.815	0.562
M × MC	3.223	4	0.806	1.537	0.201
T × MC	0.571	6	0.095	0.182	0.981
M × T × MC	3.178	12	0.265	0.505	0.905
Error	37.733	72	0.524		
Total	69,247.155	108			
Corrected Total	51.639	107			
Note: R^2^ = 0.269, adjusted R^2^ = −0.086					
Total amino acid content (%)					
Corrected Model	96.493	35	2.757	6.538	0.000
Intercept	68,033.048	1	68,033.048	161,340.717	0.000
Drying method (M)	21.588	2	10.794	25.598	0.000
Drying temperature (T)	4.743	3	1.581	3.749	0.015
Initial moisture content (MC)	18.573	2	9.286	22.023	0.000
M × T	5.357	6	0.893	2.117	0.062
M × MC	22.187	4	5.547	13.154	0.000
T × MC	10.941	6	1.824	4.325	0.001
M × T × MC	13.104	12	1.092	2.590	0.006
Error	30.360	72	0.422		
Total	68,159.902	108			
Corrected Total	126.854	107			
Note: R^2^ = 0.761, adjusted R^2^ = 0.644					

**Table 5:** Effect of drying method on the nutritional quality of peanuts.

Drying Method	Protein/%	Fat/%	Oleic Acid/%	Linoleic Acid/%	Total Amino Acids/%
20–35 °C					
MSID	25.09 ± 0.76 ^a^	47.41 ± 0.75 ^a^	25.12 ± 0.99 ^a^	48.11 ± 1.48 ^a^	25.58 ± 0.65 ^a^
HAD	25.90 ± 0.24 ^a^	46.46 ± 0.59 ^a^	25.49 ± 0.99 ^a^	47.01 ± 1.47 ^a^	23.09 ± 0.56 ^b^
RF-HAD	24.91 ± 1.41 ^a^	46.09 ± 1.09 ^a^	24.67 ± 2.03 ^a^	46.83 ± 1.40 ^a^	25.93 ± 1.17 ^a^
20–45 °C					
MSID	25.59 ± 0.23 ^ab^	48.36 ± 0.14 ^a^	26.30 ± 0.22 ^a^	46.87 ± 0.86 ^b^	25.34 ± 0.22 ^a^
HAD	25.67 ± 0.26 ^a^	48.19 ± 0.28 ^a^	27.48 ± 0.65 ^a^	47.94 ± 0.84 ^ab^	23.06 ± 0.89 ^b^
RF-HAD	25.13 ± 0.19 ^b^	47.18 ± 1.37 ^a^	25.88 ± 4.28 ^a^	50.29 ± 2.43 ^a^	25.10 ± 0.15 ^a^
20–55 °C					
MSID	25.22 ± 0.19 ^a^	49.26 ± 0.51 ^a^	31.23 ± 0.82 ^a^	44.92 ± 0.40 ^b^	25.45 ± 0.17 ^a^
HAD	25.76 ± 0.46 ^a^	49.45 ± 0.42 ^a^	29.87 ± 0.86 ^a^	46.97 ± 1.33 ^ab^	23.30 ± 0.73 ^b^
RF-HAD	25.49 ± 0.68 ^a^	48.98 ± 0.77 ^a^	30.29 ± 3.14 ^a^	48.42 ± 1.73 ^a^	23.66 ± 0.53 ^b^
20–60 °C					
MSID	25.45 ± 0.90 ^a^	49.95 ± 0.56 ^ab^	30.11 ± 0.53 ^a^	44.35 ± 0.24 ^b^	25.39 ± 0.76 ^a^
HAD	25.64 ± 0.05 ^a^	50.34 ± 0.20 ^a^	30.98 ± 1.21 ^a^	44.69 ± 0.86 ^b^	23.50 ± 0.50 ^b^
RF-HAD	25.48 ± 1.02 ^a^	48.48 ± 1.27 ^b^	28.50 ± 1.60 ^a^	48.45 ± 0.84 ^a^	25.38 ± 0.86 ^a^
25–35 °C					
MSID	25.67 ± 0.55 ^a^	45.59 ± 0.47 ^b^	24.95 ± 0.33 ^a^	49.60 ± 0.43 ^a^	25.42 ± 0.99 ^a^
HAD	25.29 ± 0.26 ^a^	47.74 ± 0.63 ^a^	23.68 ± 1.01 ^a^	44.94 ± 0.70 ^b^	25.43 ± 0.22 ^a^
RF-HAD	25.38 ± 0.43 ^a^	46.40 ± 0.11 ^b^	20.40 ± 1.20 ^b^	49.66 ± 0.59 ^a^	25.00 ± 0.39 ^a^
25–45 °C					
MSID	25.18 ± 0.99 ^a^	47.23 ± 0.39 ^b^	26.13 ± 0.42 ^a^	48.73 ± 0.43 ^a^	25.20 ± 0.38 ^a^
HAD	24.90 ± 1.01 ^a^	47.99 ± 0.90 ^ab^	24.92 ± 0.19 ^b^	45.24 ± 1.80 ^b^	25.08 ± 0.89 ^a^
RF-HAD	25.67 ± 0.65 ^a^	49.20 ± 0.82 ^a^	26.38 ± 0.70 ^a^	47.75 ± 0.64 ^a^	23.28 ± 0.54 ^b^
25–55 °C					
MSID	25.18 ± 0.78 ^a^	47.70 ± 1.03 ^b^	27.53 ± 0.73 ^a^	48.31 ± 0.47 ^a^	25.60 ± 0.46 ^a^
HAD	25.67 ± 0.95 ^a^	48.97 ± 0.64 ^ab^	28.42 ± 2.60 ^a^	44.49 ± 0.60 ^b^	25.86 ± 0.81 ^a^
RF-HAD	25.65 ± 0.37 ^a^	49.53 ± 0.57 ^b^	27.13 ± 1.28 ^a^	47.92 ± 1.05 ^a^	25.20 ± 0.28 ^a^
25–60 °C					
MSID	24.98 ± 1.21 ^a^	49.23 ± 0.99 ^a^	28.91 ± 0.62 ^a^	46.62 ± 1.59 ^a^	25.77 ± 1.10 ^a^
HAD	25.46 ± 0.68 ^a^	49.33 ± 0.50 ^a^	27.84 ± 0.66 ^a^	42.58 ± 0.69 ^b^	24.97 ± 0.14 ^a^
RF-HAD	25.79 ± 1.23 ^a^	49.93 ± 1.36 ^a^	28.46 ± 2.07 ^a^	47.29 ± 1.33 ^a^	25.04 ± 1.09 ^a^
30–35 °C					
MSID	24.53 ± 0.69 ^a^	46.40 ± 0.81 ^a^	24.50 ± 1.03 ^ab^	49.39 ± 0.61 ^a^	25.37 ± 0.59 ^a^
HAD	24.97 ± 0.37 ^a^	47.24 ± 0.64 ^a^	24.72 ± 0.96 ^a^	46.84 ± 0.66 ^b^	25.37 ± 0.29 ^a^
RF-HAD	25.23 ± 1.16 ^a^	45.61 ± 0.90 ^a^	21.71 ± 1.99 ^b^	49.55 ± 1.49 ^a^	24.84 ± 0.97 ^a^
30–45 °C					
MSID	25.42 ± 0.47 ^a^	47.21 ± 0.29 ^a^	25.00 ± 1.21 ^a^	50.12 ± 1.19 ^a^	26.15 ± 0.41 ^a^
HAD	24.45 ± 0.85 ^a^	47.98 ± 0.17 ^a^	24.92 ± 1.22 ^a^	45.94 ± 0.85 ^b^	24.72 ± 0.75 ^b^
RF-HAD	25.58 ± 0.20 ^a^	45.27 ± 0.89 ^b^	23.65 ± 1.25 ^a^	47.10 ± 0.93 ^b^	24.99 ± 0.18 ^b^
30–55 °C					
MSID	25.32 ± 0.31 ^a^	48.67 ± 0.65 ^a^	27.89 ± 1.46 ^ab^	49.32 ± 1.07 ^a^	26.86 ± 0.22 ^a^
HAD	24.99 ± 0.16 ^a^	49.03 ± 0.62 ^a^	28.42 ± 0.68 ^a^	44.38 ± 1.11 ^b^	25.37 ± 0.17 ^b^
RF-HAD	25.35 ± 1.19 ^a^	45.87 ± 1.29 ^b^	24.96 ± 2.26 ^b^	46.40 ± 0.90 ^b^	26.81 ± 1.03 ^a^
30–60 °C					
MSID	24.49 ± 0.86 ^a^	48.69 ± 1.31 ^ab^	29.31 ± 1.61 ^a^	48.22 ± 0.39 ^a^	25.31 ± 0.72 ^b^
HAD	25.27 ± 0.43 ^a^	50.05 ± 0.77 ^a^	28.95 ± 1.30 ^a^	44.01 ± 1.41 ^b^	24.58 ± 0.41 ^b^
RF-HAD	25.45 ± 0.32 ^a^	47.12 ± 0.48 ^b^	24.15 ± 0.97 ^b^	45.53 ± 1.08 ^b^	26.55 ± 0.24 ^a^

Note: The different lowercase letters indicate a statistically significant difference at *p* < 0.05.

**Table 6:** Effect of drying methods on the textural characteristics of peanuts.

Drying Method	Hardness/N	Adhesiveness/N×mm	Cohesiveness	Springiness/mm	Gumminess/N	Chewiness/mJ
20–35 °C						
MSID	52.48 ± 2.45 ^b^	0.39 ± 0.05 ^a^	0.08 ± 0.01 ^a^	0.39 ± 0.03 ^ab^	4.26 ± 0.66 ^a^	1.67 ± 0.38 ^ab^
HAD	48.96 ± 5.54 ^b^	0.20 ± 0.05 ^b^	0.07 ± 0.04 ^a^	0.30 ± 0.12 ^b^	3.48 ± 1.85 ^a^	1.23 ± 0.93 ^b^
RF-HAD	61.18 ± 3.22 ^a^	0.12 ± 0.02 ^c^	0.09 ± 0.01 ^a^	0.42 ± 0.02 ^a^	5.28 ± 0.72 ^a^	2.20 ± 0.27 ^a^
20–45 °C						
MSID	63.18 ± 9.55 ^a^	0.39 ± 0.03 ^a^	0.08 ± 0.01 ^b^	0.38 ± 0.03 ^b^	5.02 ± 0.62 ^b^	2.06 ± 0.46 ^b^
HAD	51.60 ± 4.35 ^b^	0.11 ± 0.01 ^b^	0.08 ± 0.00 ^b^	0.39 ± 0.01 ^b^	4.20 ± 0.40 ^b^	1.64 ± 0.19 ^b^
RF-HAD	73.48 ± 5.99 ^a^	0.34 ± 0.06 ^a^	0.12 ± 0.01 ^a^	0.56 ± 0.04 ^a^	8.88 ± 1.40 ^a^	5.01 ± 1.17 ^a^
20–55 °C						
MSID	54.94 ± 7.80 ^c^	0.24 ± 0.05 ^a^	0.10 ± 0.01 ^b^	0.44 ± 0.04 ^b^	5.16 ± 0.86 ^b^	2.29 ± 0.58 ^b^
HAD	64.52 ± 3.82 ^b^	0.14 ± 0.02 ^b^	0.08 ± 0.02 ^b^	0.46 ± 0.03 ^b^	5.18 ± 0.93 ^b^	2.41 ± 0.52 ^b^
RF-HAD	82.62 ± 5.24 ^a^	0.13 ± 0.01 ^b^	0.12 ± 0.01 ^a^	0.64 ± 0.05 ^a^	10.24 ± 0.99 ^a^	6.59 ± 1.04 ^a^
20–60 °C						
MSID	68.92 ± 4.32 ^b^	0.37 ± 0.04 ^a^	0.10 ± 0.01 ^b^	0.49 ± 0.04 ^b^	7.24 ± 1.22 ^b^	3.57 ± 0.89 ^b^
HAD	66.90 ± 6.39 ^b^	0.11 ± 0.02 ^b^	0.10 ± 0.01 ^b^	0.50 ± 0.05 ^b^	6.38 ± 1.01 ^b^	3.15 ± 0.74 ^b^
RF-HAD	108.92 ± 14.83 ^a^	0.16 ± 0.04 ^b^	0.12 ± 0.02 ^a^	0.73 ± 0.04 ^a^	13.66 ± 3.96 ^a^	10.04 ± 3.46 ^a^
25–35 °C						
MSID	46.16 ± 7.64 ^ab^	0.15 ± 0.03 ^a^	0.07 ± 0.02 ^b^	0.36 ± 0.06 ^b^	3.10 ± 0.66 ^b^	1.15 ± 0.38 ^b^
HAD	48.90 ± 2.24 ^a^	0.15 ± 0.03 ^a^	0.10 ± 0.02 ^a^	0.50 ± 0.11 ^a^	4.60 ± 0.97 ^a^	2.39 ± 1.01 ^a^
RF-HAD	40.00 ± 2.64 ^b^	0.13 ± 0.03 ^a^	0.11 ± 0.01 ^a^	0.52 ± 0.04 ^a^	4.40 ± 0.51 ^a^	2.30 ± 0.43 ^a^
25–45 °C						
MSID	61.44 ± 2.23 ^a^	0.31 ± 0.04 ^a^	0.08 ± 0.01 ^b^	0.41 ± 0.04 ^c^	5.20 ± 0.35 ^a^	2.10 ± 0.13 ^b^
HAD	62.78 ± 4.10 ^a^	0.16 ± 0.03 ^b^	0.09 ± 0.01 ^ab^	0.50 ± 0.04 ^b^	5.42 ± 0.36 ^a^	2.71 ± 0.35 ^b^
RF-HAD	58.61 ± 3.02 ^a^	0.12 ± 0.02 ^b^	0.10 ± 0.02 ^a^	0.69 ± 0.07 ^a^	6.08 ± 0.96 ^a^	4.22 ± 0.86 ^a^
25–55 °C						
MSID	61.14 ± 6.39 ^b^	0.13 ± 0.02 ^ab^	0.09 ± 0.03 ^a^	0.42 ± 0.04 ^b^	5.28 ± 1.59 ^b^	2.27 ± 0.78 ^b^
HAD	57.54 ± 5.57 ^b^	0.11 ± 0.01 ^b^	0.11 ± 0.02 ^a^	0.48 ± 0.04 ^b^	6.16 ± 0.70 ^b^	2.97 ± 0.53 ^b^
RF-HAD	83.60 ± 1.20 ^a^	0.13 ± 0.01 ^a^	0.11 ± 0.01 ^a^	0.60 ± 0.04 ^a^	9.42 ± 1.13 ^a^	5.61 ± 0.76 ^a^
25–60 °C						
MSID	72.54 ± 7.40 ^b^	0.15 ± 0.01 ^b^	0.09 ± 0.01 ^b^	0.47 ± 0.06 ^b^	6.78 ± 1.20 ^b^	3.18 ± 0.87 ^b^
HAD	64.50 ± 8.95 ^b^	0.25 ± 0.03 ^a^	0.10 ± 0.01 ^ab^	0.44 ± 0.03 ^b^	6.48 ± 1.10 ^b^	2.85 ± 0.42 ^b^
RF-HAD	93.00 ± 10.14 ^a^	0.12 ± 0.02 ^b^	0.12 ± 0.01 ^a^	0.58 ± 0.02 ^a^	10.88 ± 1.88 ^a^	6.26 ± 0.95 ^a^
30–35 °C						
MSID	85.68 ± 4.65 ^a^	0.27 ± 0.05 ^b^	0.11 ± 0.01 ^a^	0.58 ± 0.03 ^a^	9.24 ± 1.33 ^a^	5.38 ± 0.51 ^a^
HAD	65.19 ± 3.19 ^b^	0.50 ± 0.05 ^a^	0.10 ± 0.01 ^a^	0.45 ± 0.05 ^b^	6.65 ± 0.54 ^b^	3.00 ± 0.47 ^b^
RF-HAD	45.09 ± 4.54 ^c^	0.09 ± 0.01 ^c^	0.11 ± 0.03 ^a^	0.39 ± 0.08 ^b^	4.67 ± 1.64 ^c^	1.95 ± 0.96 ^b^
30–45 °C						
MSID	49.50 ± 3.85 ^c^	0.13 ± 0.02 ^b^	0.11 ± 0.03 ^a^	0.51 ± 0.08 ^a^	5.22 ± 1.48 ^b^	2.72 ± 1.24 ^b^
HAD	80.67 ± 8.42 ^a^	0.57 ± 0.05 ^a^	0.12 ± 0.04 ^a^	0.50 ± 0.04 ^a^	10.06 ± 3.33 ^a^	4.99 ± 1.69 ^a^
RF-HAD	61.90 ± 2.77 ^b^	0.14 ± 0.04 ^b^	0.10 ± 0.02 ^a^	0.47 ± 0.04 ^a^	6.46 ± 1.60 ^b^	3.04 ± 0.75 ^ab^
30–55 °C						
MSID	62.14 ± 4.23 ^b^	0.12 ± 0.01 ^b^	0.11 ± 0.01 ^a^	0.49 ± 0.04 ^ab^	6.84 ± 1.00 ^b^	3.41 ± 0.67 ^b^
HAD	74.76 ± 8.10 ^a^	0.54 ± 0.08 ^a^	0.11 ± 0.02 ^a^	0.44 ± 0.01 ^b^	8.35 ± 2.22 ^ab^	3.70 ± 1.02 ^ab^
RF-HAD	75.88 ± 3.27 ^a^	0.11 ± 0.01 ^b^	0.12 ± 0.01 ^a^	0.53 ± 0.09 ^a^	9.15 ± 0.54 ^a^	4.90 ± 0.96 ^a^
30–60 °C						
MSID	92.50 ± 7.54 ^a^	0.12 ± 0.03 ^b^	0.12 ± 0.03 ^a^	0.56 ± 0.04 ^a^	10.94 ± 3.15 ^ab^	6.29 ± 2.11 ^a^
HAD	78.87 ± 7.83 ^b^	0.55 ± 0.06 ^a^	0.11 ± 0.01 ^a^	0.54 ± 0.05 ^a^	9.05 ± 1.43 ^b^	4.91 ± 1.02 ^a^
RF-HAD	97.26 ± 10.59 ^a^	0.14 ± 0.03 ^b^	0.16 ± 0.05 ^a^	0.54 ± 0.08 ^a^	15.85 ± 5.92 ^a^	8.54 ± 3.48 ^a^

Note: The different lowercase letters indicate a statistically significant difference at *p* < 0.05.

**Table 7:** Effect of moisture content on the textural characteristics of peanuts.

Moisture Content/%	Hardness/N	Adhesiveness/N×mm	Cohesiveness	Springiness/mm	Gumminess/N	Chewiness/mJ
MSID-35 °C						
20	52.48 ± 2.46 ^b^	0.39 ± 0.05 ^a^	0.08 ± 0.01 ^b^	0.39 ± 0.03 ^b^	4.26 ± 0.66 ^b^	1.67 ± 0.38 ^b^
25	46.16 ± 7.64 ^b^	0.15 ± 0.03 ^c^	0.07 ± 0.02 ^b^	0.36 ± 0.06 ^b^	3.10 ± 0.66 ^b^	1.15 ± 0.37 ^b^
30	85.68 ± 4.65 ^a^	0.27 ± 0.05 ^b^	0.11 ± 0.01 ^a^	0.58 ± 0.03 ^a^	9.24 ± 1.33 ^a^	5.38 ± 0.51 ^a^
MSID-45 °C						
20	63.18 ± 9.55 ^a^	0.39 ± 0.03 ^a^	0.08 ± 0.01 ^a^	0.38 ± 0.03 ^b^	5.02 ± 0.62 ^a^	2.06 ± 0.46 ^a^
25	61.44 ± 2.23 ^a^	0.31 ± 0.04 ^b^	0.08 ± 0.01 ^a^	0.41 ± 0.04 ^b^	5.20 ± 0.35 ^a^	2.10 ± 0.13 ^a^
30	49.50 ± 3.85 ^b^	0.13 ± 0.02 ^c^	0.11 ± 0.03 ^a^	0.51 ± 0.08 ^a^	5.22 ± 1.48 ^a^	2.72 ± 1.24 ^a^
MSID-55 °C						
20	54.94 ± 7.80 ^a^	0.24 ± 0.05 ^a^	0.10 ± 0.01 ^a^	0.44 ± 0.04 ^ab^	5.16 ± 0.86 ^a^	2.29 ± 0.58 ^b^
25	61.14 ± 6.39 ^a^	0.13 ± 0.02 ^b^	0.09 ± 0.03 ^a^	0.42 ± 0.04 ^b^	5.28 ± 1.59 ^a^	2.27 ± 0.78 ^b^
30	62.14 ± 4.23 ^a^	0.12 ± 0.01 ^b^	0.11 ± 0.01 ^a^	0.49 ± 0.04 ^a^	6.84 ± 1.00 ^a^	3.41 ± 0.67 ^a^
MSID-60 °C						
20	68.92 ± 4.32 ^b^	0.37 ± 0.04 ^a^	0.10 ± 0.01 ^a^	0.49 ± 0.04 ^b^	7.24 ± 1.22 ^b^	3.57 ± 0.89 ^b^
25	72.54 ± 7.40 ^b^	0.15 ± 0.01 ^b^	0.09 ± 0.01 ^a^	0.47 ± 0.06 ^b^	6.78 ± 1.20 ^b^	3.18 ± 0.87 ^b^
30	92.50 ± 7.54 ^a^	0.12 ± 0.03 ^b^	0.12 ± 0.03 ^a^	0.57 ± 0.04 ^a^	10.94 ± 3.15 ^a^	6.29 ± 2.11 ^a^
HAD-35 °C						
20	48.96 ± 5.54 ^b^	0.20 ± 0.05 ^b^	0.07 ± 0.03 ^a^	0.30 ± 0.12 ^b^	3.48 ± 1.85 ^b^	1.23 ± 0.93 ^b^
25	48.90 ± 2.24 ^b^	0.15 ± 0.03 ^b^	0.09 ± 0.02 ^a^	0.50 ± 0.11 ^a^	4.60 ± 0.97 ^b^	2.39 ± 1.01 ^ab^
30	65.19 ± 3.19 ^a^	0.50 ± 0.05 ^a^	0.10 ± 0.01 ^a^	0.45 ± 0.05 ^ab^	6.65 ± 0.54 ^aa^	3.00 ± 0.47 ^a^
HAD-45 °C						
20	51.60 ± 4.35 ^c^	0.11 ± 0.01 ^b^	0.08 ± 0.01 ^b^	0.39 ± 0.01 ^b^	4.20 ± 0.40 ^b^	1.64 ± 0.19 ^b^
25	62.78 ± 4.10 ^b^	0.16 ± 0.01 ^b^	0.09 ± 0.01 ^b^	0.50 ± 0.04 ^a^	5.42 ± 0.36 ^b^	2.71 ± 0.35 ^b^
30	80.67 ± 8.42 ^a^	0.57 ± 0.05 ^a^	0.12 ± 0.04 ^a^	0.50 ± 0.04 ^a^	10.06 ± 3.33 ^a^	4.99 ± 1.69 ^a^
HAD-55 °C						
20	64.52 ± 3.83 ^b^	0.14 ± 0.02 ^b^	0.08 ± 0.02 ^b^	0.46 ± 0.03 ^a^	5.18 ± 0.93 ^b^	2.41 ± 0.52 ^b^
25	57.54 ± 5.58 ^b^	0.11 ± 0.01 ^b^	0.11 ± 0.02 ^ab^	0.48 ± 0.04 ^a^	6.16 ± 0.70 ^ab^	2.97 ± 0.53 ^ab^
30	74.76 ± 8.10 ^a^	0.54 ± 0.08 ^a^	0.11 ± 0.02 ^a^	0.44 ± 0.01 ^a^	8.35 ± 2.22 ^a^	3.70 ± 1.02 ^a^
HAD-60 °C						
20	66.90 ± 6.39 ^ab^	0.11 ± 0.02 ^c^	0.10 ± 0.01 ^a^	0.50 ± 0.05 ^ab^	6.38 ± 1.01 ^b^	3.15 ± 0.74 ^b^
25	64.50 ± 8.94 ^b^	0.25 ± 0.03 ^b^	0.10 ± 0.01 ^a^	0.44 ± 0.03 ^b^	6.48 ± 1.10 ^b^	2.85 ± 0.42 ^b^
30	78.87 ± 7.83 ^a^	0.55 ± 0.06 ^a^	0.11 ± 0.01 ^a^	0.54 ± 0.05 ^a^	9.05 ± 1.43 ^a^	4.91 ± 1.02 ^a^
RF-HAD-35 °C						
20	61.18 ± 3.22 ^a^	0.12 ± 0.02 ^a^	0.09 ± 0.01 ^a^	0.42 ± 0.02 ^b^	5.28 ± 0.72 ^a^	2.20 ± 0.27 ^a^
25	40.00 ± 2.64 ^b^	0.13 ± 0.03 ^a^	0.11 ± 0.01 ^a^	0.52 ± 0.04 ^a^	4.40 ± 0.51 ^a^	2.30 ± 0.43 ^a^
30	42.09 ± 4.54 ^b^	0.09 ± 0.01 ^b^	0.11 ± 0.03 ^a^	0.39 ± 0.08 ^b^	4.67 ± 1.64 ^a^	1.95 ± 0.96 ^a^
RF-HAD-45 °C						
20	73.48 ± 5.99 ^a^	0.34 ± 0.06 ^a^	0.12 ± 0.01 ^a^	0.56 ± 0.04 ^b^	8.88 ± 1.40 ^a^	5.01 ± 1.17 ^a^
25	58.61 ± 3.02 ^b^	0.12 ± 0.02 ^b^	0.10 ± 0.02 ^a^	0.69 ± 0.08 ^a^	6.08 ± 0.96 ^b^	4.22 ± 0.86 ^ab^
30	61.90 ± 2.77 ^b^	0.14 ± 0.04 ^b^	0.10 ± 0.02 ^a^	0.47 ± 0.04 ^c^	6.46 ± 1.60 ^b^	3.04 ± 0.75 ^b^
RF-HAD-55 °C						
20	82.62 ± 5.24 ^a^	0.13 ± 0.01 ^a^	0.12 ± 0.01 ^a^	0.64 ± 0.05 ^a^	10.24 ± 0.99 ^a^	6.59 ± 1.04 ^a^
25	83.60 ± 1.20 ^a^	0.13 ± 0.01 ^a^	0.11 ± 0.01 ^a^	0.60 ± 0.04 ^ab^	9.42 ± 1.13 ^a^	5.61 ± 0.76 ^ab^
30	75.88 ± 3.27 ^b^	0.11 ± 0.01 ^b^	0.12 ± 0.01 ^a^	0.53 ± 0.09 ^b^	9.15 ± 0.54 ^a^	4.90 ± 0.96 ^a^
RF-HAD-60 °C						
20	108.92 ± 14.83 ^a^	0.16 ± 0.04 ^a^	0.12 ± 0.02 ^a^	0.73 ± 0.04 ^a^	13.66 ± 3.96 ^a^	10.04 ± 3.46 ^a^
25	93.00 ± 10.14 ^a^	0.12 ± 0.02 ^a^	0.12 ± 0.01 ^a^	0.58 ± 0.02 ^b^	10.88 ± 1.88 ^a^	6.26 ± 0.95 ^a^
30	97.27 ± 10.59 ^a^	0.14 ± 0.03 ^a^	0.16 ± 0.05 ^a^	0.54 ± 0.08 ^b^	15.85 ± 5.92 ^a^	8.54 ± 3.48 ^a^

Note: The different lowercase letters indicate a statistically significant difference at *p* < 0.05.

**Table 8:** Three-way ANOVA results for the textural characteristics of peanuts.

Source	Type III Sum of Squares	df	Mean Square	F-Value	*p*-Value
Hardness					
Corrected Model	45,521.446	35	1300.613	32.113	0.000
Intercept	809,598.053	1	809,598.053	19,989.552	0.000
Drying method (M)	3406.805	2	1703.402	42.058	0.000
Drying temperature (T)	18,959.617	3	6319.872	156.042	0.000
Initial moisture content (MC)	2846.033	2	1423.016	35.135	0.000
M × T	9127.442	6	1521.240	37.561	0.000
M × MC	4981.190	4	1245.298	30.747	0.000
T × MC	1411.620	6	235.270	5.809	0.000
M × T × MC	4788.739	12	399.062	9.853	0.000
Error	5832.153	144	40.501		
Total	860,951.651	180			
Corrected Total	51,353.598	179			
Note: R^2^ = 0.886, adjusted R^2^ = 0.859					
Cohesiveness					
Corrected Model	0.060	35	0.002	4.253	0.000
Intercept	1.892	1	1.892	4717.297	0.000
Drying method (M)	0.016	2	0.008	20.341	0.000
Drying temperature (T)	0.011	3	0.004	9.450	0.000
Initial moisture content (MC)	0.015	2	0.008	19.215	0.000
M × T	0.002	6	0.000	0.880	0.511
M × MC	0.004	4	0.001	2.731	0.031
T × MC	0.002	6	0.000	0.730	0.626
M × T × MC	0.008	12	0.001	1.733	0.065
Error	0.058	144	0.000		
Total	2.010	180			
Corrected Total	0.117	179			
Note: R^2^ = 0.508, adjusted R^2^ = 0.389					
Adhesiveness					
Corrected Model	3.621	35	0.103	83.095	0.000
Intercept	8.698	1	8.698	6985.786	0.000
Drying method (M)	0.589	2	0.295	236.657	0.000
Drying temperature (T)	0.112	3	0.037	29.964	0.000
Initial moisture content (MC)	0.394	2	0.197	158.310	0.000
M × T	0.107	6	0.018	14.262	0.000
M × MC	2.103	4	0.526	422.348	0.000
T × MC	0.041	6	0.007	5.437	0.000
M × T × MC	0.275	12	0.023	18.408	0.000
Error	0.179	144	0.001		
Total	12.498	180			
Corrected Total	3.800	179			
Note: R^2^ = 0.953, adjusted R^2^ = 0.941					
Springiness					
Corrected Model	1.437	35	0.041	14.183	0.000
Intercept	43.336	1	43.336	14,973.295	0.000
Drying method (M)	0.376	2	0.188	65.037	0.000
Drying temperature (T)	0.243	3	0.081	27.940	0.000
Initial moisture content (MC)	0.027	2	0.013	4.607	0.011
M × T	0.112	6	0.019	6.466	0.000
M × MC	0.380	4	0.095	32.820	0.000
T × MC	0.185	6	0.031	10.650	0.000
M × T × MC	0.114	12	0.009	3.275	0.000
Error	0.417	144	0.003		
Total	45.189	180			
Corrected Total	1.853	179			
Note: R^2^ = 0.775, adjusted R^2^ = 0.720					
Gumminess					
Corrected Model	1434.853	35	40.996	12.865	0.000
Intercept	9050.433	1	9050.433	2840.187	0.000
Drying method (M)	247.796	2	123.898	38.881	0.000
Drying temperature (T)	519.462	3	173.154	54.339	0.000
Initial moisture content (MC)	194.729	2	97.365	30.555	0.000
M × T	239.093	6	39.849	12.505	0.000
M × MC	98.512	4	24.628	7.729	0.000
T × MC	36.560	6	6.093	1.912	0.083
M × T × MC	98.701	12	8.225	2.581	0.004
Error	458.865	144	3.187		
Total	10,944.150	180			
Corrected Total	1893.718	179			
Note: R^2^ = 0.758, adjusted R^2^ = 0.699					
Chewiness					
Corrected Model	719.566	35	20.559	14.924	0.000
Intercept	2445.429	1	2445.429	1775.166	0.000
Drying method (M)	168.743	2	84.371	61.246	0.000
Drying temperature (T)	227.030	3	75.677	54.935	0.000
Initial moisture content (MC)	49.113	2	24.556	17.826	0.000
M × T	128.952	6	21.492	15.601	0.000
M × MC	82.076	4	20.519	14.895	0.000
T × MC	29.400	6	4.900	3.557	0.003
M × T × MC	34.253	12	2.854	2.072	0.022
Error	198.371	144	1.378		
Total	3363.367	180			
Corrected Total	917.937	179			
Note: R^2^ = 0.784, adjusted R^2^ = 0.731					

**Table 9:** Effect of drying temperature on acid value and peroxide value.

Moisture Content/%	20		25		30	
Temperature/°C	Acid Value	Peroxide Value	Acid Value	Peroxide Value	Acid Value	Peroxide Value
MSID						
35	0.24 ± 0.05 ^c^	0.04 ± 0.01 ^a^	0.23 ± 0.00 ^b^	0.03 ± 0.01 ^b^	0.28 ± 0.02 ^b^	0.01 ± 0.00 ^b^
45	0.34 ± 0.00 ^b^	0.05 ± 0.02 ^a^	0.30 ± 0.10 ^b^	0.03 ± 0.01 ^b^	0.35 ± 0.01 ^b^	0.01 ± 0.00 ^b^
55	0.48 ± 0.01 ^a^	0.05 ± 0.00 ^a^	0.33 ± 0.02 ^b^	0.04 ± 0.00 ^ab^	0.42 ± 0.05 ^b^	0.04 ± 0.00 ^a^
60	0.55 ± 0.03 ^a^	0.07 ± 0.01 ^a^	0.56 ± 0.02 ^a^	0.05 ± 0.00 ^a^	0.66 ± 0.09 ^a^	0.04 ± 0.00 ^a^
HAD						
35	0.31 ± 0.01 ^b^	0.04 ± 0.01 ^a^	0.32 ± 0.04 ^c^	0.01 ± 0.00 ^c^	0.28 ± 0.10 ^b^	0.01 ± 0.00 ^d^
45	0.38 ± 0.02 ^b^	0.05 ± 0.01 ^a^	0.44 ± 0.03 ^b^	0.03 ± 0.00 ^c^	0.30 ± 0.03 ^b^	0.02 ± 0.00 ^c^
55	0.58 ± 0.09 ^a^	0.05 ± 0.01 ^a^	0.60 ± 0.01 ^a^	0.05 ± 0.00 ^b^	0.39 ± 0.02 ^ab^	0.03 ± 0.00 ^b^
60	0.68 ± 0.03 ^a^	0.05 ± 0.00 ^a^	0.61 ± 0.06 ^a^	0.07 ± 0.01 ^a^	0.49 ± 0.00 ^a^	0.04 ± 0.00 ^a^
RF-HAD						
35	0.26 ± 0.02 ^b^	0.04 ± 0.01 ^ab^	0.28 ± 0.01 ^c^	0.03 ± 0.00 ^c^	0.35 ± 0.02 ^c^	0.01 ± 0.00 ^d^
45	0.42 ± 0.06 ^ab^	0.04 ± 0.01 ^b^	0.35 ± 0.10 ^bc^	0.03 ± 0.00 ^bc^	0.52 ± 0.02 ^bc^	0.03 ± 0.00 ^c^
55	0.53 ± 0.09 ^a^	0.06 ± 0.02 ^ab^	0.52 ± 0.09 ^b^	0.04 ± 0.01 ^b^	0.65 ± 0.06 ^ab^	0.04 ± 0.00 ^b^
60	0.58 ± 0.05 ^a^	0.07 ± 0.01 ^a^	0.78 ± 0.14 ^a^	0.07 ± 0.00 ^a^	0.90 ± 0.18 ^a^	0.05 ± 0.00 ^a^

Note: The different lowercase letters indicate a statistically significant difference at *p* < 0.05.

**Table 10:** Effect of moisture content on acid value and peroxide value.

Temperature/°C	35		45		55		60	
Moisture Content/%	Acid Value	Peroxide Value	Acid Value	Peroxide Value	Acid Value	Peroxide Value	Acid Value	Peroxide Value
MSID								
20	0.24 ± 0.05 ^a^	0.04 ± 0.01 ^a^	0.34 ± 0.00 ^a^	0.05 ± 0.02 ^a^	0.48 ± 0.01 ^a^	0.05 ± 0.00 ^a^	0.55 ± 0.03 ^a^	0.07 ± 0.01 ^a^
25	0.23 ± 0.00 ^a^	0.03 ± 0.01 ^b^	0.30 ± 0.10 ^a^	0.03 ± 0.01 ^a^	0.33 ± 0.02 ^b^	0.04 ± 0.01 ^b^	0.56 ± 0.02 ^a^	0.05 ± 0.00 ^ab^
30	0.28 ± 0.02 ^a^	0.01 ± 0.00 ^b^	0.35 ± 0.01 ^a^	0.01 ± 0.00 ^a^	0.42 ± 0.05 ^a^	0.04 ± 0.00 ^b^	0.66 ± 0.09 ^a^	0.04 ± 0.00 ^b^
HAD								
20	0.31 ± 0.01 ^a^	0.04 ± 0.01 ^a^	0.38 ± 0.02 ^ab^	0.05 ± 0.01 ^a^	0.58 ± 0.09 ^a^	0.05 ± 0.01 ^a^	0.68 ± 0.03 ^a^	0.05 ± 0.00 ^b^
25	0.32 ± 0.04 ^a^	0.01 ± 0.00 ^b^	0.44 ± 0.03 ^a^	0.03 ± 0.00 ^b^	0.60 ± 0.01 ^a^	0.05 ± 0.00 ^a^	0.61 ± 0.06 ^a^	0.07 ± 0.01 ^a^
30	0.28 ± 0.10 ^a^	0.01 ± 0.00 ^b^	0.30 ± 0.03 ^b^	0.02 ± 0.00 ^b^	0.39 ± 0.02 ^b^	0.03 ± 0.00 ^b^	0.49 ± 0.00 ^b^	0.04 ± 0.00 ^b^
RF-HAD								
20	0.26 ± 0.23 ^b^	0.04 ± 0.01 ^a^	0.42 ± 0.06 ^a^	0.04 ± 0.01 ^a^	0.53 ± 0.10 ^a^	0.06 ± 0.02 ^a^	0.58 ± 0.05 ^b^	0.07 ± 0.01 ^a^
25	0.28 ± 0.03 ^b^	0.03 ± 0.00 ^a^	0.35 ± 0.10 ^a^	0.03 ± 0.00 ^a^	0.52 ± 0.09 ^a^	0.04 ± 0.01 ^a^	0.78 ± 0.01 ^ab^	0.07 ± 0.00 ^a^
30	0.35 ± 0.22 ^a^	0.01 ± 0.00 ^b^	0.52 ± 0.02 ^a^	0.03 ± 0.00 ^a^	0.65 ± 0.06 ^a^	0.04 ± 0.00 ^a^	0.90 ± 0.18 ^a^	0.04 ± 0.00 ^b^

Note: The different lowercase letters indicate a statistically significant difference at *p* < 0.05.

**Table 11:** Effect of drying method on acid value and peroxide value.

Moisture Content/%	20		25		30	
Drying Method	Acid Value	Peroxide Value	Acid Value	Peroxide Value	Acid Value	Peroxide Value
35 °C						
MSID	0.24 ± 0.05 ^a^	0.04 ± 0.01 ^a^	0.23 ± 000 ^b^	0.03 ± 0.01 ^a^	0.28 ± 0.02 ^q^	0.01 ± 0.00 ^q^
HAD	0.31 ± 0.01 ^a^	0.04 ± 0.01 ^a^	0.32 ± 0.04 ^a^	0.01 ± 0.00 ^b^	0.28 ± 0.10 ^q^	0.01 ± 0.00 ^q^
RF-HAD	0.26 ± 0.02 ^a^	0.04 ± 0.01 ^a^	0.28 ± 0.00 ^ab^	0.03 ± 0.00 ^a^	0.35 ± 0.02 ^q^	0.01 ± 0.00 ^q^
45 °C						
MSID	0.34 ± 0.00 ^a^	0.05 ± 0.02 ^a^	0.30 ± 0.10 ^a^	0.03 ± 0.01 ^a^	0.35 ± 0.01 ^b^	0.02 ± 0.00 ^b^
HAD	0.38 ± 0.02 ^a^	0.05 ± 0.01 ^a^	0.44 ± 0.03 ^a^	0.03 ± 0.00 ^a^	0.30 ± 0.03 ^b^	0.02 ± 0.00 ^b^
RF-HAD	0.42 ± 0.06 ^a^	0.04 ± 0.01 ^a^	0.35 ± 0.10 ^a^	0.03 ± 0.00 ^a^	0.52 ± 0.02 ^a^	0.03 ± 0.00 ^a^
55 °C						
MSID	0.48 ± 0.01 ^a^	0.05 ± 0.00 ^a^	0.33 ± 0.02 ^b^	0.04 ± 0.01 ^a^	0.42 ± 0.05 ^b^	0.04 ± 0.00 ^a^
HAD	0.58 ± 0.10 ^a^	0.05 ± 0.01 ^a^	0.60 ± 0.01 ^a^	0.05 ± 0.00 ^a^	0.39 ± 0.02 ^b^	0.03 ± 0.00 ^b^
RF-HAD	0.53 ± 0.09 ^a^	0.06 ± 0.02 ^a^	0.52 ± 0.09 ^a^	0.04 ± 0.01 ^a^	0.65 ± 0.06 ^a^	0.04 ± 0.00 ^a^
60 °C						
MSID	0.55 ± 0.03 ^b^	0.07 ± 0.01 ^a^	0.56 ± 0.02 ^b^	0.05 ± 0.00 ^a^	0.66 ± 0.09 ^ab^	0.04 ± 0.00 ^ab^
HAD	0.68 ± 0.03 ^a^	0.05 ± 0.00 ^b^	0.61 ± 0.06 ^b^	0.07 ± 0.01 ^a^	0.49 ± 0.00 ^b^	0.04 ± 0.00 ^b^
RF-HAD	0.58 ± 0.05 ^b^	0.07 ± 0.01 ^a^	0.78 ± 0.01 ^a^	0.07 ± 0.00 ^a^	0.90 ± 1.76 ^a^	0.05 ± 0.00 ^a^

Note: The different lowercase letters indicate a statistically significant difference at *p* < 0.05.

**Table 12:** Three-way ANOVA results for acid value and peroxide value.

Source	Type III Sum of Squares	df	Mean Square	F-Value	*p*-Value
Acid value					
Corrected Model	1.858	35	0.053	16.215	0.000
Intercept	14.694	1	14.694	4487.385	0.000
Drying method (M)	0.165	2	0.082	25.188	0.000
Drying temperature (T)	1.318	3	0.439	134.173	0.000
Initial moisture content (MC)	0.007	2	0.004	1.138	0.332
M × T	0.057	6	0.010	2.925	0.020
M × MC	0.209	4	0.052	15.950	0.000
T × MC	0.025	6	0.004	1.261	0.300
M × T × MC	0.077	12	0.006	1.953	0.060
Error	0.118	36	0.003		
Total	16.670	72			
Corrected Total	1.976	71			
Note: R^2^ = 0.940, adjusted R^2^ = 0.882					
Peroxide value					
Corrected Model	0.365	35	0.010	101.082	0.000
Intercept	0.180	1	0.180	1741.515	0.000
Drying method (M)	0.014	2	0.007	69.135	0.000
Drying temperature (T)	0.069	3	0.023	223.146	0.000
Initial moisture content (MC)	0.044	2	0.022	212.817	0.000
M × T	0.054	6	0.009	86.870	0.000
M × MC	0.035	4	0.009	84.538	0.000
T × MC	0.049	6	0.008	79.696	0.000
M × T × MC	0.100	12	0.008	80.582	0.000
Error	0.004	36	0.000		
Total	0.549	72			
Corrected Total	0.369	71			
Note: R^2^ = 0.990, adjusted R^2^ = 0.980					

## Data Availability

The original contributions presented in this study are included in the article. Further inquiries can be directed to the corresponding author.

## Abbreviations

The following abbreviations are used in this manuscript:

MSID	Mid- and short-wave infrared drying
HAD	Hot air drying
RF-HAD	Radio frequency-hot air combined drying
AV	Acid value
POV	Peroxide value
